# Construction and Validation of a Novel Prognostic Signature of Idiopathic Pulmonary Fibrosis by Identifying Subtypes Based on Genes Related to 7-Methylguanosine Modification

**DOI:** 10.3389/fgene.2022.890530

**Published:** 2022-06-09

**Authors:** Tao Huang, Wei-Ying He

**Affiliations:** ^1^ Department of Cardiothoracic Vascular Surgery, The Affiliated Hospital of Youjiang Medical University for Nationalities, Baise, China; ^2^ The First Clinical Medical College, The First Affiliated Hospital of Guangxi Medical University, Nanning, China

**Keywords:** m7G, prognosis, signature, immunology, marker

## Abstract

**Background:** Idiopathic pulmonary fibrosis (IPF) is the interstitial lung disease with the highest incidence and mortality. The lack of specific markers results in limited treatment methods for IPF patients. Numerous prognostic signatures represented effective indexes in predicting the survival of patients in various diseases; however, little is investigated on their application in IPF.

**Methods:** This study attempted to explore the clinical markers suitable for IPF by constructing a prognostic signature from the perspective of 7-methylguanosine (m7G). An m7G-related prognostic signature (m7GPS) was established based on the discovery cohort with the LASSO algorithm and was verified by internal and external validation cohorts. The area under the curve (AUC) values were utilized to assess the accuracy of m7GPS in predicting the prognosis of IPF patients and the ability of m7GPS in screening IPF patients. Kaplan-Meier curves and Cox regression analyses were used to identify the relationship of m7GPS with the prognosis of IPF individuals. Enrichment analyses, CIBERSORT algorithm, and weighted gene co-expression network analysis were applied to explore the underlying mechanisms and correlation of m7GPS in IPF.

**Results:** The two m7G regulatory genes can divide IPF into subtypes 1 and 2, and subtype 2 demonstrated a poor prognosis for IPF patients (*p* < 0.05). For the first time in this field, the m7GPS was constructed. m7GPS made it feasible to predict the 1–5 years survival status of IPF patients (AUC = 0.730–0.971), and it was an independent prognostic risk factor for IPF patients (hazard ratio > 1, *p* < 0.05). The conspicuous ability of m7GPS to screen IPF patients from the healthy was also revealed by an AUC value of 0.960. The roles of m7GPS in IPF may link to inflammation, immune response, and immune cell levels. Seven genes (*CYR61*, etc.) were identified as hub genes of m7GPS in IPF. Three drugs (ZM447439-1050, AZD1332-1463, and Ribociclib-1632) were considered sensitive to patients with high m7GPS risk scores.

**Conclusion:** This study developed a novel m7GPS, which is a reliable indicator for predicting the survival status of IPF patients and is identified as an effective marker for prognosis and screening of IPF patients.

## 1 Introduction

Idiopathic pulmonary fibrosis (IPF) is the most common interstitial lung disease with unknown etiology, mainly characterized by chronic and progressive pulmonary fibrosis. In the United States and Europe, the annual incidence of IPF is 5–20 per 100,000 (Nalysnyk et al., 2012; Ley and Collard, 2013). Lung transplantation is considered the only approach for curing and improving the survival of IPF patients. However, this treatment was constrained by the limited number of donors and the unoptimistic acceptability of recipients (George et al., 2019). Currently, only pirfenidone and nintedanib have been approved by Food and Drug Administration for clinical application of IPF patients. Nevertheless, although these two drugs can slightly reduce the number of people with lung function deterioration, they can neither significantly prevent the disease progression nor reduce the mortality of IPF patients (King et al., 2014b; Richeldi et al., 2014; Raghu et al., 2015), making IPF the disease with the worst prognosis in pulmonary interstitial disease. The predicted survival of most IPF patients after diagnosis is 2–3 years, and the 5-year survival rate of the disease is lower than 40% (King et al., 2001; Navaratnam et al., 2011; King et al., 2014a). Additionally, the lack of markers to identify the disease progression of IPF patients further poses challenges for the diagnosis and treatment of IPF. Therefore, there is an urgent need to explore specific and effective markers to identify the prognostic risk of IPF and provide the potential reference value for individualized treatment of IPF patients.

RNA methylations are common epigenetic modifications in eukaryotes, including N1-methyladenosine (m1A), N6-methyladenosine (m6A), 5-methylcytosine (m5C), and 7-methylguanosine (m7G). The application of RNA methylation-related prognostic signatures for promoting the individualized treatment of human diseases has received considerable critical attention. For example, a prognostic signature constructed based on m6A-modified long noncoding RNAs was identified as a risk factor and predictor of the prognosis of patients with low-grade gliomas (Nguyen et al., 2022). An m1A-, m5C-, and m6A-related prognostic signature was considered the underlying biomarker of cutaneous melanoma (Wu et al., 2021). However, most current research on RNA methylation regulation-related prognosis signatures only focuses on tumor diseases. Little is known about whether the RNA methylation signature is applied to human non-tumor diseases, including IPF. Thus, novel attempts are needed to fill up the vacancy.

For the first time in this field, this study established an m7G-related prognostic signature (m7GPS) for IPF based on the discovery cohort and verified it by the internal and external validation cohorts. Further, the study also explored the prognostic value and potential molecular mechanisms of m7G regulatory genes (m7GRGs) in IPF, which may promote the understanding of m7GRGs in IPF and reveal clinical markers for predicting the prognosis of patients with IPF.

## 2 Materials and Methods

### The Collection of Cohorts and Their Clinical Information

**FIGURE 1:**
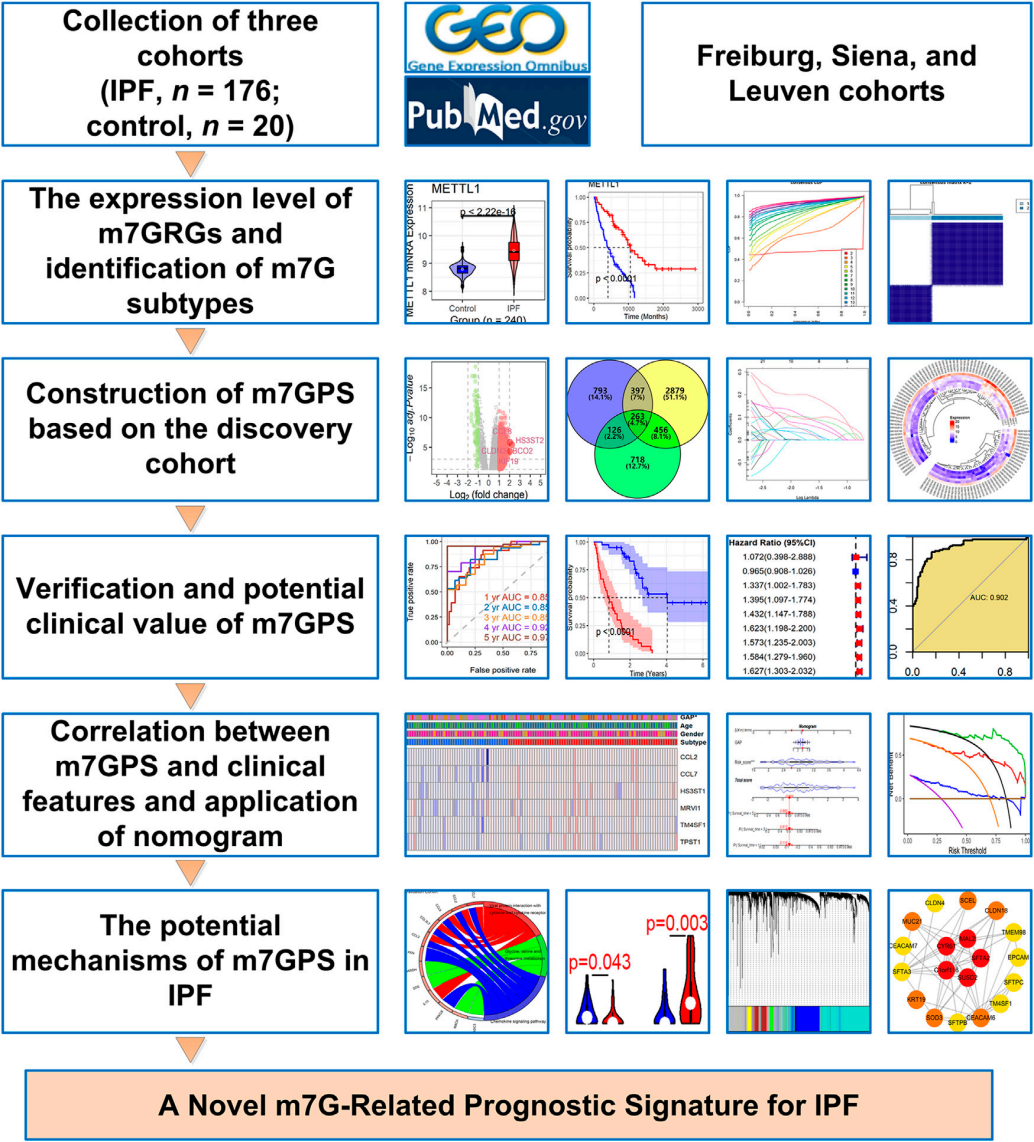
The design and main results of this study. IPF, idiopathic pulmonary fibrosis; m7GPS, m7G-related prognostic signature.

Three IPF-related cohorts and the corresponding clinical data were collected from the Gene Expression Omnibus database, containing the Freiburg, Siena, and Leuven cohorts, all of which belong to the series GSE70866. Given that data of the Freiburg cohort and Siena cohort were detected with the same platform GPL14550, they were combined as the Freiburg-Siena cohort for further analysis. The gene expression levels of Freiburg-Siena and Leuven cohorts were normalized with log_2_ (*x* + 1). The two cohorts included multiple bronchoalveolar lavage samples obtained from IPF patients (*n* = 176) and normal healthy individuals (*n* = 20) (Figure 1). Considering the apparent difference in the number of samples between IPF (*n* of Freiburg-Siena = 112) and healthy (*n* of Freiburg-Siena = 20) groups, the SMOTE (synthetic minority oversampling technique) algorithm was used to balance the sample size. This process was completed by the “DMwR” package for R (v4.1.0), and the target samples of both the IPF group and the healthy group (for the new Freiburg-Siena cohort) were set to 120. The new data produced based on SMOTE were used to explore the expression difference and identification effect of m7GRGs between the IPF and healthy groups.

### The Identification of m7G Subtypes

Three m7GRGs, *BUD23*, *METTL1*, and *RNMT*, were collected from the literature survey (Ouyang et al., 2021). *METTL1* and *RNMT* were included in both the Freiburg-Siena and Leuven cohorts and thus were further analyzed in this study. Based on the Freiburg-Siena cohort (the samples were more than the Leuven cohort), the average method was used to identify various m7G subtypes related to m7GRGs, with clustering by the *k*-means algorithm and describing the distance metric by euclidean. The *k* values were set as 2–20, and the optimal *k* value was determined by the cumulative distribution function plot. The identification of m7G subtypes was performed by utilizing the “ConsensuClusterPlus” package (Wilkerson and Hayes, 2010) in R (v4.1.0).

### The Construction of m7GPS

The gene expression data of the Freiburg-Siena cohort was used to select differentially expressed genes (DEGs) in various m7G subtypes by the threshold of the absolute value of log_2_ (fold change) > 1 and adjusted *p*-value < 0.05, and the step was finished with the help of “limma” package (Ritchie et al., 2015). Based on data from both the Freiburg-Siena and Leuven datasets, prognosis-related genes (PRGs) were chosen by the *p* values of univariate Cox regression analysis less than 0.05. Candidate genes for constructing the m7GPS were identified from the intersection of DEGs and PRGs. With the “sample” algorithm in R (v4.1.0), IPF patients from the Freiburg-Siena cohort were randomly classified into the discovery cohort (70%) and internal validation cohort (30%). The Leuven cohort was identified as the external validation cohort.

Multiple methods have been used in medical prognostic signatures, such as the least absolute shrinkage and selection operator (LASSO) Cox regression and Random Forest. Considering that LASSO Cox regression was more common in this field (Supplementary Figure S1), it was used to construct the m7GPS in our study. The expression levels of candidate genes, survival status, and survival time data of the discovery cohort were applied to establish the m7GPS (consisting of specific genes) using the LASSO Cox regression. In addition to two parameters (family = “cox” and maxit = 1,000), all parameters of the “glmnet” function (for fitting the signature) and the “cv.glmnet” function (for validating the signature by using *k*-fold cross) were set by default (Friedman et al., 2010; Simon et al., 2011).

### The Verification of m7GPS and Its Clinical Value

Using time-dependent receiver operating characteristic (ROC) curves (Robin et al., 2011) produced by the “timeROC” package, the accuracy of m7GPS in predicting the prognosis was assessed through the area under the curve (AUC) values of the discovery, internal validation, and external validation cohorts. A high AUC value (ranging from 0 to 1) represented the conspicuous accuracy of the m7GPS in predicting the survival of IPF patients. Moreover, confusion matrixes (calculated by the “caret” package) and F1 score (ranging from 0 to 1) were also utilized to assess the precision of the m7GPS. For the F1 score, the increasing value indicated a more conspicuous classification performance of m7GPS. The relationship of m7GPS with the prognosis of IPF individuals was evaluated by both the Kaplan-Meier curves (with log-rank test) and Cox regression analyses. For Cox regression analyses, an indicator (e.g., a gene) with a hazard ratio (HR) of more than 1 indicated a poor prognosis for IPF patients. AUC values were also applied to explore the ability of m7GPS and its constitutive genes in screening IPF patients.

A nomogram was constructed based on prognosis-related factors identified from univariate Cox analysis, where the survival probabilities of IPF patients can be predicted. Calibration curves were utilized to detect the accuracy of the nomogram in predicting the survival condition of patients. Decision curve analysis (DCA) (Vickers and Elkin, 2006) was applied to evaluate the net benefits for IPF patients in predicting survival rates based on the nomogram.

### The Potential Mechanisms of m7GPS and Its Underlying Target Drugs

Differential expression genes (DENGs) between the high-risk group and low-risk group were identified for exploring the potential molecular functions and KEGG (Kyoto Encyclopedia of Genes and Genomes) (Kanehisa et al., 2017) signaling pathways of m7GPS in IPF. The calculation processes were carried out by the “limma,” “clusterProfiler,” and “GOplot” packages (Yu et al., 2012; Walter et al., 2015). The CIBERSORT (Newman et al., 2019) algorithm was utilized to predict the composition of 22 types of immune cells for the discovery, internal validation, and external validation cohorts. Wilcoxon rank-sum tests were exerted to disclose the difference in infiltration levels of immune cells between the high-risk group and low-risk group.

The weighted gene co-expression network analysis (WGCNA) determined candidate hub genes of m7GPS. In detail, the median absolute deviation (MAD) of all genes in the discovery cohort was calculated, and genes with a MAD value of more than one were screened for performing WGCNA. The steps of WGCNA were as follows: 1) the top 5,000 genes with the highest MAD values were divided into various modules related to the m7GPS risk score using the “WGCNA” package (Langfelder and Horvath, 2008); 2) The scale-free topology fitting index R^2^ was set as 0.85 and identified the minimum soft threshold; 3) the correlations between genes in each module and m7GPS risk score were evaluated by gene significance and module membership; 4) the genes of the essential module were output for selecting hub genes. Subsequently, hub genes of m7GPS were identified in Cytoscape (v3.9.0) based on the “degree” algorithm. The same steps were also utilized for internal and external validation cohorts.

The “oncoPredict” package (Maeser et al., 2021) was applied to predict potential drugs (from the GDSC2 database) sensitive to IPF patients with high risk scores (more than the median value of all risk scores in the corresponding cohort). The drug sensitivity was assessed by half-limiting dose values.

### Statistical Analysis

The expression difference of m7GRGs between IPF and control groups was identified by Wilcoxon rank-sum test. The test was also utilized to evaluate clinical characteristics—ages, genders, GAP (gender-age-physiologic) stages, and subtypes—between high and low risk score IPF groups. All the analyses in this study were carried out in R (v4.1.0) and Cytoscape (v3.9.0). The design of this study is shown in Figure 1.

## 3 Results

### The Expression Level of m7GRGs and the Identification of m7G Subtype

**FIGURE 2:**
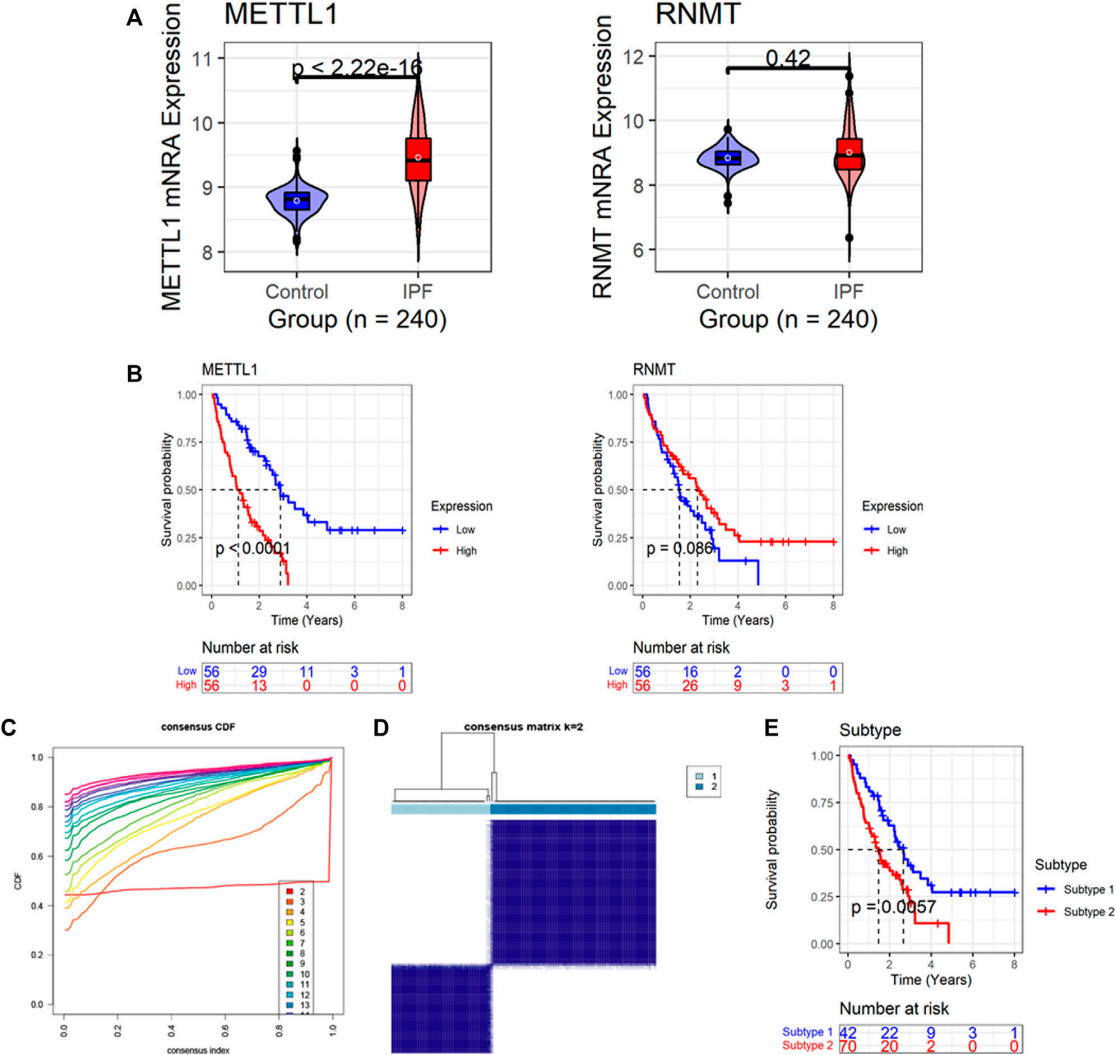
The expression and prognostic significance of m7G regulatory genes (m7GRGs) in IPF. **(A)** Differences in expression of m7GRGs between IPF and normal bronchoalveolar lavage cells (synthetic minority oversampling technique data were used in this panel); the *p*-value is calculated based on Wilcoxon rank-sum test. **(B)** The association of m7GRGs expression levels with the prognosis of IPF patients; the *p*-value is calculated based on log-rank test. **(C)** The cumulative distribution function plot demonstrates that among 2–20, the optimal *k* value of subtypes equals 2. **(D)** Heatmaps of the consensus matrices for *k* = 2; the panel shows that patients can be significantly divided into two subtypes (42 patients in the subtype 1, and 70 patients in the subtype 2). **(E)** The different prognosis between the two subtypes; the *p*-value is calculated based on log-rank test.

According to the new data based on SMOTE (*n* = 240), the expression level of *METTL1* in the IPF group was higher than that in the normal group (*p* < 0.05); in contrast, the expression of *RNMT* between the two groups was not statistically different (*p* = 0.42) (Figure 2A). IPF patients with high *METTL1* expression had an unfavorable prognosis—shorter survival (*p* < 0.05, Figure 2B). Based on the Freiburg-Siena cohort, despite no statistical significance, patients with upregulated *RNMT* expression tended to have an optimistic prognosis—more prolonged survival (*p* = 0.086, Figure 2B). Based on the expression of the two m7GRGs—*METTL1* and *RNMT*, IPF patients in the Freiburg-Siena cohort were classified into two subtypes (the optimal *k* = 2) by the unsupervised clustering algorithm (Figures 2C,D). Subtype 1 and subtype 2 contained 42 and 70 IPF patients, respectively (Supplementary Table S1). The analysis of the Kaplan-Meier curve showed that the clinical prognosis of these two m7G subtypes was different, and IPF patients in subtype 2 had more unfavorable survival (*p* < 0.05, Figure 2E).

### The Construction of m7GPS

**FIGURE 3:**
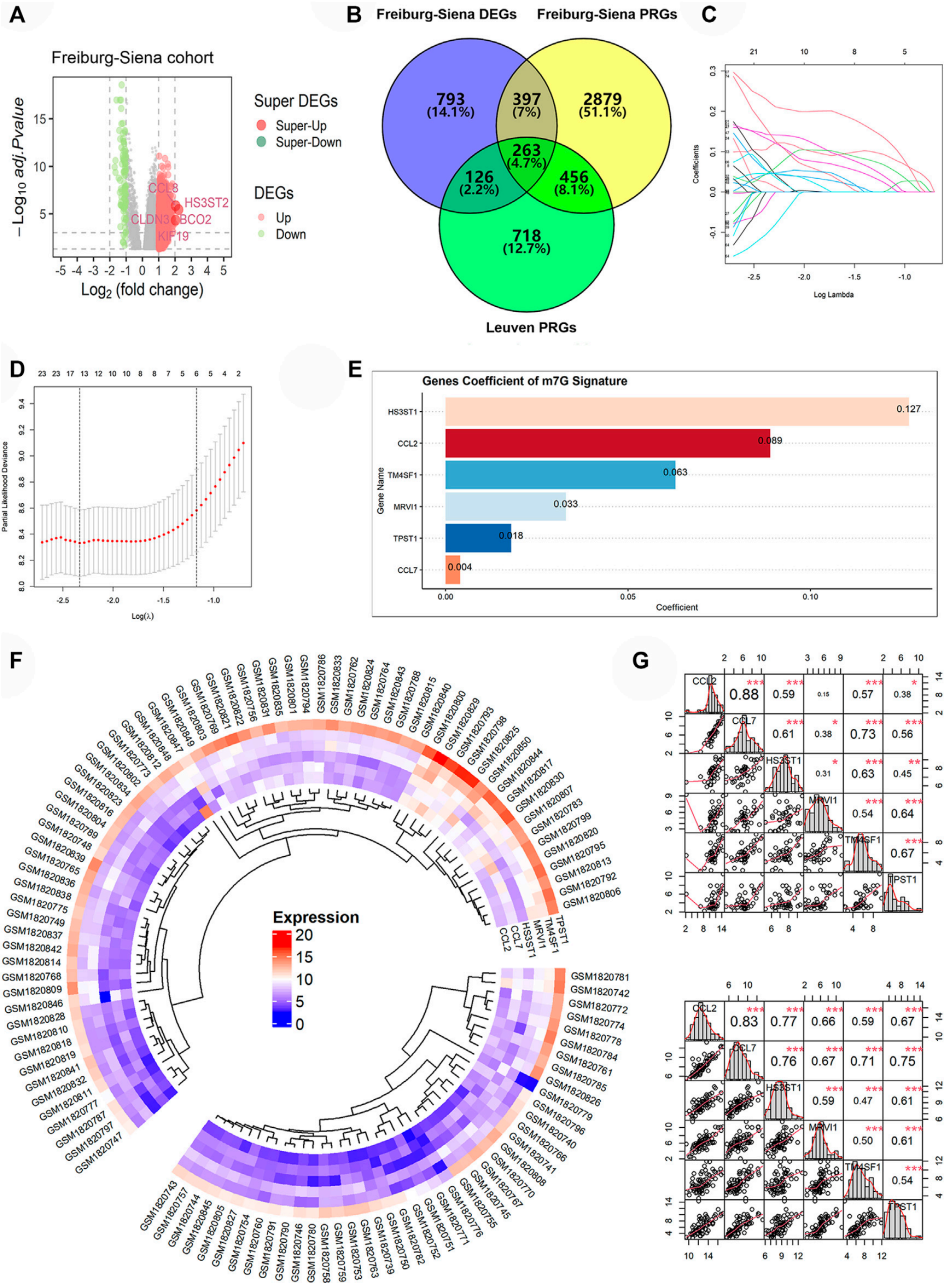
The construction of m7GPS. **(A,B)** Two hundred and sixty-three genes were identified as candidate genes for m7GPS; DEGs, differentially expressed genes; PRGs, prognosis-related genes. **(C)** Least absolute shrinkage and selection operator (LASSO) coefficient profiles of the 263 candidate genes. **(D)** The coefficient profile plot was produced against the log (lambda) sequence in the LASSO model, and the optimal parameter (lambda) was selected when the gene number equals 6. **(E)** Coefficients of six genes in m7GPS. **(F)** Differential expression levels of six genes between the subtype 1 group (the smaller fan-shaped region) and the subtype 2 group (the larger fan-shaped region); the “complete” clustering method of “phearmap” package was used in this panel. **(G)** Co-expression relationship of six genes in the subtype 1 group (the top panel) and the subtype 2 group (the bottom panel); Spearman correlation coefficient was used in this panel; *p < 0.05; **p < 0.01; ***p < 0.001.

Based on the Freiburg-Siena cohort, there were 1,579 DEGs between the various subtypes (subtype 2 versus subtype 1), including 1,490 upregulated expression genes and 89 downregulated expression genes (Figure 3A). Using univariate Cox regression analyses, 3,995 and 1,563 PRGs were identified from the Freiburg-Siena and Leuven queues, respectively. Two hundred and sixty-three m7GPS candidate genes were obtained by the intersection of DEGs and PRGs (Figure 3B), and they would be utilized to establish the m7GPS subsequently. Seventy-nine (70%) patients in the Freiburg-Siena cohort were randomly assigned to the discovery cohort, and the remaining 33 patients (30%) were assigned to the internal validation cohort (Supplementary Tables S2, S3). The ratio of survival status (alive or dead) between the two cohorts was balanced, and such a condition can also be seen for GAP stages (*p* > 0.05; Supplementary Table S4), suggesting the random grouping of the discovery and internal validation cohorts was unbiased for IPF severity. Another queue, Leuven, was defined as the external validation cohort (Supplementary Table S5). Although there was a statistically significant difference in GAP stages between the discovery and external validation cohorts, the distribution of both 1-year and 2-year survival/death ratios was balanced (Supplementary Table S4). Moreover, considering that the discovery cohort and the external validation cohort came from different countries and cities (Prasse et al., 2019) and that it is meaningful to verify the effectiveness of the m7GPS by cohorts of different countries and cities, the external validation cohort was conserved in this study.

Based on the discovery cohort and the LASSO Cox regression algorithm, the m7GPS containing six candidate genes—*CCL2*, *CCL7*, *HS3ST1*, *MRVI1*, *TM4SF1*, and *TPST1*—was constructed (Figures 3C,D). The coefficients of the six genes for calculating m7GPS risk scores can be seen in Figure 3E. The expression level of the six genes in subtype 2 was higher than that in subtype 1 (Figure 3F), and the expression level of the six genes in subtypes 1 and 2 were significantly positively correlated, respectively (Figure 3G), suggesting the rationality of m7GPS subtypes to some extent. Moreover, the positive or negative relationship between m7GPS and the two m7GRGs (*METTL1* and *RNMT*) can be seen in Supplementary Figure S2, implying the association of m7GPS with m7G.

### Accuracy and Potential Clinical Value of m7GPS

**FIGURE 4:**
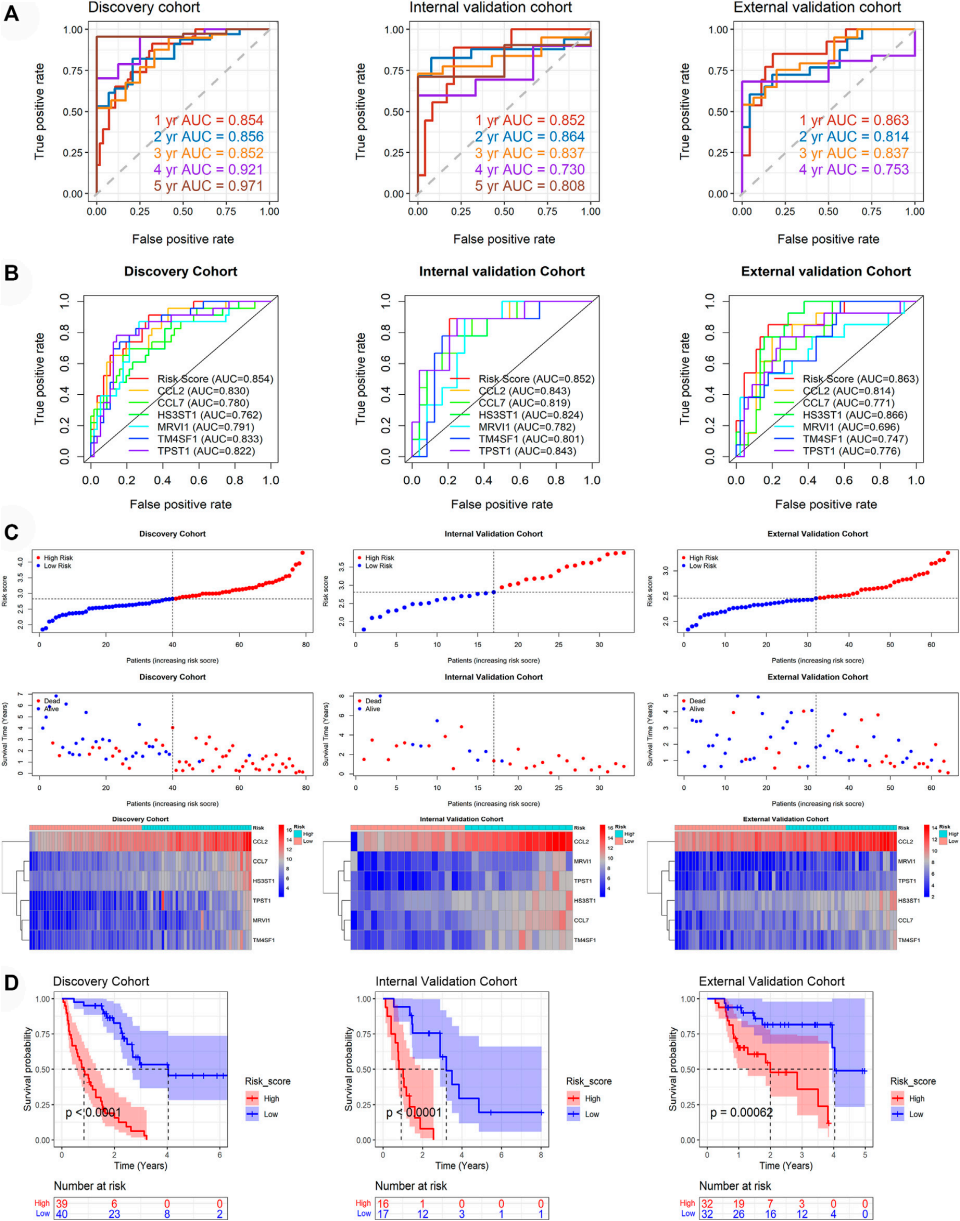
The prediction accuracy and prognostic value of m7GPS in IPF. **(A)** The accuracy of m7GPS in predicting the 1-, 3-, and 5-year survival status (alive or dead) of IPF patients. **(B)** The accuracy of m7GPS and single gene in predicting the 1-survival status of IPF patients. **(C)** Risk plots and heatmaps of the expression profile of m7GPS and clinical characteristics in the discovery, internal validation, and external validation cohorts. **(D)** The different prognosis between the high-risk (patients with a risk score less than the median value of the cohort) and low-risk (the other patients) groups of the discovery, internal validation, and external validation cohorts; the *p*-value is calculated based on log-rank test.

By analyzing the discovery cohort, m7GPS can highly accurately predict the 1–5 years survival status of IPF patients (AUC = 0.852–0.971; Figure 4A). This conclusion was confirmed by the internal and external validation cohorts (AUC = 0.730–0.864; Figure 4A). Notably, the sample number of the external validation cohort was not enough to calculate the 5-year AUC. The confusion matrixes verified the reliability of the m7GPS, and F1 scores (0.611–0.839) also supported the conspicuous precision of the m7GPS in predicting the survival status of IPF patients (Supplementary Table S6). In addition, m7GPS can predict the 1-year survival status of IPF more accurately than almost all single genes of m7GPS (Figure 4B).

**FIGURE 5:**
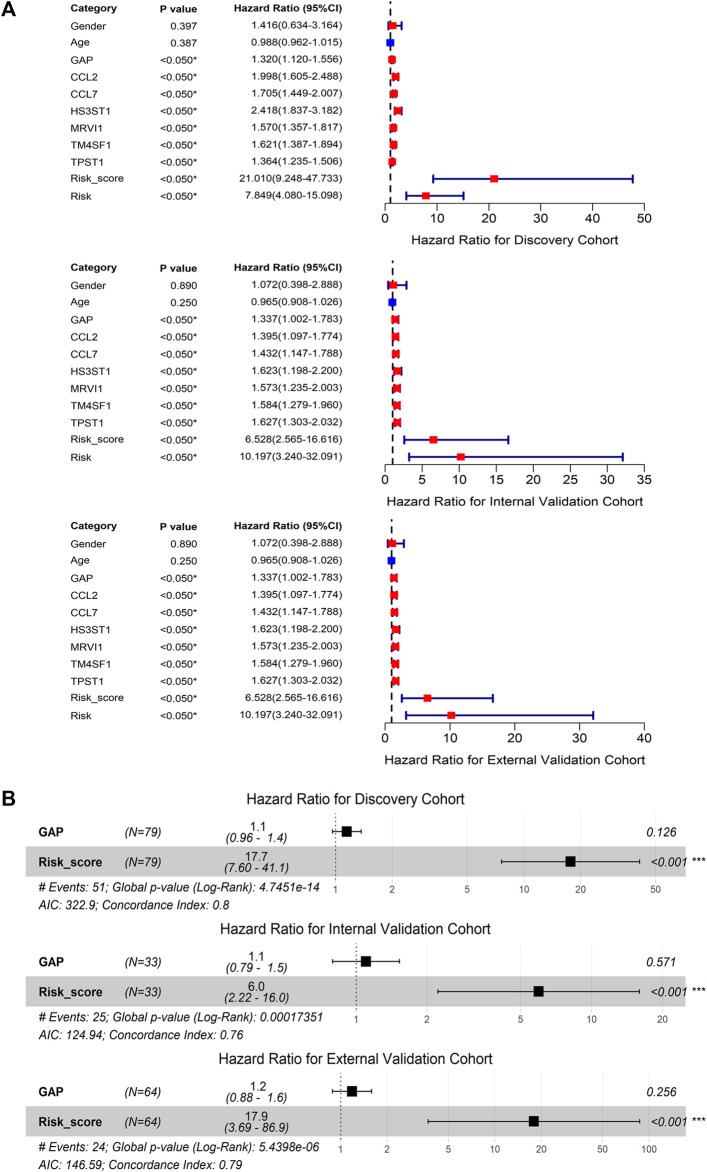
The prognostic value of m7GPS and clinical characteristics in IPF. **(A)** Univariate Cox regression analysis. **(B)** Multivariate Cox regression analysis; ***p < 0.001. GAP, gender-age-physiologic variables.

The discovery, internal validation, and external validation cohorts consistently indicated that IPF deaths were more common in patients with m7GPS high risk scores; IPF individuals with a high m7GPS risk score had elevated expression levels of six genes (Figure 4C). Compared with patients with low risk scores, the data of discovery, internal validation, and external validation cohorts supported that IPF patients with high risk scores had a shorter survival time (Figure 4D). Furthermore, the univariate Cox regression analysis of the three cohorts showed that GAP, risk score, and the six genes (*CCL2*, *CCL7*, *HS3ST1*, *MRVI1*, *TM4SF1*, and *TPST1*) were related to the poor prognosis of IPF individuals (HR > 1, *p* < 0.05; Figure 5A). Multivariate Cox regression analyses of GAP and risk score demonstrated that the m7GPS risk score was an independent risk factor for the prognosis of IPF patients (HR > 1, *p* < 0.05; Figure 5B). Additionally, the concordance indexes of GAP stages combined with m7GPS were 0.76–0.80 for the discovery, internal validation, and external validation cohorts (Figure 5B), verifying the accuracy of m7GPS in predicting the prognosis of IPF patients. Although Random Forest was not utilized to establish the m7GPS, the concordance index (0.74) of the prognostic signature based on this method (using the “randomForestSRC” package) was not conspicuously less than that of the m7GPS based on LASSO Cox regression (Supplementary Figures S1B,C); this verified the feasibility of constructing prognostic signature from the perspective of m7G.

**FIGURE 6:**
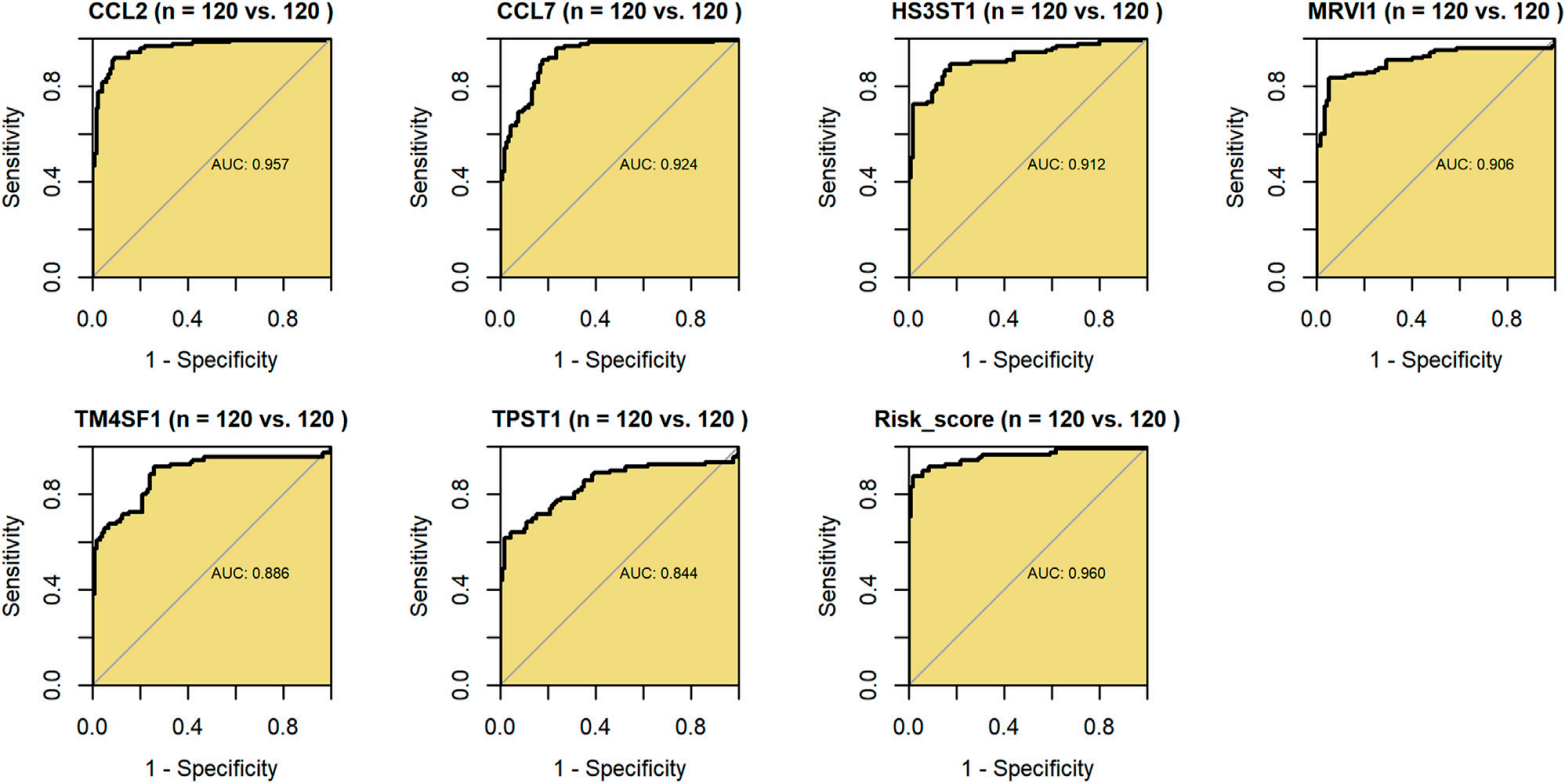
The accuracy of m7GPS and its constitutive genes in distinguishing IPF bronchoalveolar lavage and healthy lung bronchoalveolar lavage. AUC, area under the curve. Synthetic minority oversampling technique data were used in this figure.

Further, *via* ROC curves of the 240 samples based on SMOTE method, the constitutive genes (*CCL2*, *CCL7*, *HS3ST1*, *MRVI1*, *TM4SF1*, and *TPST1*) and risk score of m7GPS demonstrated conspicuous accuracy in distinguishing IPF patients and healthy individuals (AUC > 0.843, Figure 6). Compared to its constitutive genes, m7GPS had the highest accuracy in differentiating IPF patients (AUC = 0.960; Figure 6), presenting as an excellent marker in screening IPF based on bronchoalveolar lavage.

### Correlation Between m7GPS and Clinical Features and Application of Nomogram

**FIGURE 7:**
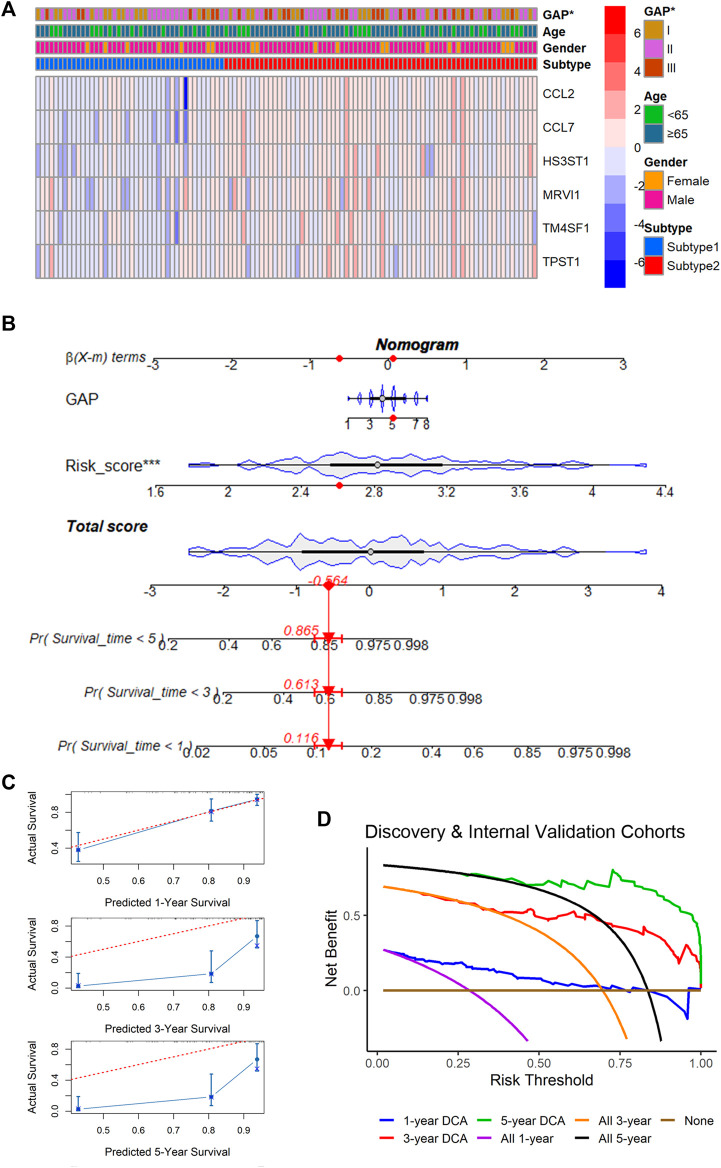
Correlation between m7GPS and clinical features and an application of the m7CPS in the nomogram. **(A)** Correlation between m7GPS and clinical features; the *p*-value is calculated based on Wilcoxon rank-sum test. **(B)** The nomogram is based on the GAP and m7GPS; the red dots in the nomogram represent the clinical features of patient GSM1820740. **(C)** Calibration curves assess the accuracy of the nomogram in predicting the survival probabilities of IPF individuals. **(D)** Decision curve analysis (DCA) demonstrates positive net benefits of the nomogram for IPF patients.

Although there was no statistical difference in ages and genders between m7G subtypes, GAP stages were statistically different among various m7G subtypes, implying the underlying clinical value (similar to GAP stages) of the m7GPS (based on m7G subtypes) (Figure 7A). To explore the potential clinical application value of m7GPS, this study constructed a nomogram based on GAP and risk score (both of which were related to the prognosis of patients in univariate Cox analysis) (Figure 7B). The 1-, 3-, and 5-year survival probabilities of IPF patients can be predicted according to the nomogram. For example, for the IPF patient (ID: GSM1820740) with GAP scores equaling to 5 and m7GPS risk scores equaling to 2.605, the predicted probability of his survival being less than 5 years, 3 years, and 1 year was 0.865, 0.613, and 0.116, respectively (Figure 7B). According to the calibration curve, the prediction accuracy of the nomogram was evaluated. The 1-year survival probability predicted by the nomogram was similar to the actual survival probability of IPF patients, although the probability of long-term survival (3- and 5-years) predicted by the nomogram was relatively inaccurate (Figure 7C). DCA revealed that compared with the non-implementation strategy and all strategies, the nomogram based on GAP and m7GPS provided net benefits for IPF patients in predicting 1-, 3-, and 5-year survival rates (Figure 7D).

### The Potential Molecular Mechanisms of and Underlying Target Drugs for m7GPS

**FIGURE 8:**
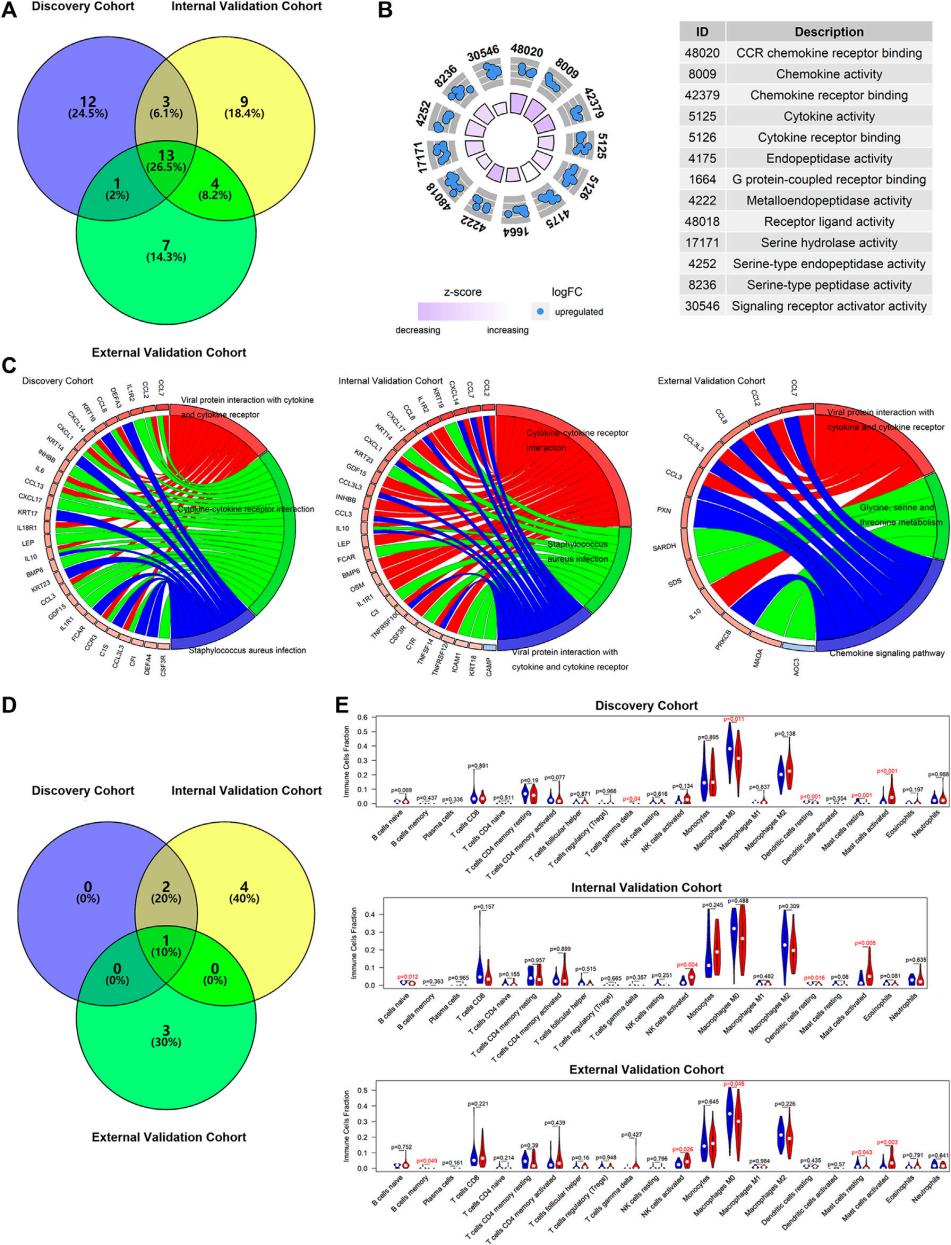
The potential molecular mechanisms of m7GPS in IPF. **(A)** Selection of overlap molecular functions of m7GPS in the three cohorts. **(B)** The panel was drawn based on the validation cohort with the overlapped molecular functions of m7GPS in the three cohorts. **(C)** KEGG signaling pathways of the m7GPS are based on the three cohorts. **(D)** Selection of KEGG signaling pathways of m7GPS in the three cohorts. **(E)** The immune cell fraction levels between patients with high- and low-risk scores; *p*-value was calculated based on Wilcoxon rank-sum test.

The DENGs between the high-risk and low-risk groups (classified based on the median risk scores in the cohort) were determined by discovery cohort data, and they were used to perform the molecular functions and KEGG analyses. The same calculations were performed for the internal and external validation cohorts. The results showed that 13 functions were screened simultaneously from three cohorts (Figure 8A), such as receptor ligand activity, signaling receptor activator activity, cytokine activity, and chemokine activity (Figure 8B). The results of the KEGG signaling pathway also suggested similar results (Figure 8C). The signaling pathway, viral protein interaction with cytokine and cytokine receptor, was found in all discovery, internal validation, and external validation cohorts (Figure 8D), suggesting that the potential molecular mechanism of m7GPS may be related to this pathway.

The data analysis results of the three cohorts showed that IPF patients with high m7GPS risk scores had increasing levels of activated mast cells (Figure 8E). In addition, increasing NK cells and decreasing M0 macrophages cells and dendritic cells were detected in patients with high risk scores (Figure 8E).

**FIGURE 9:**
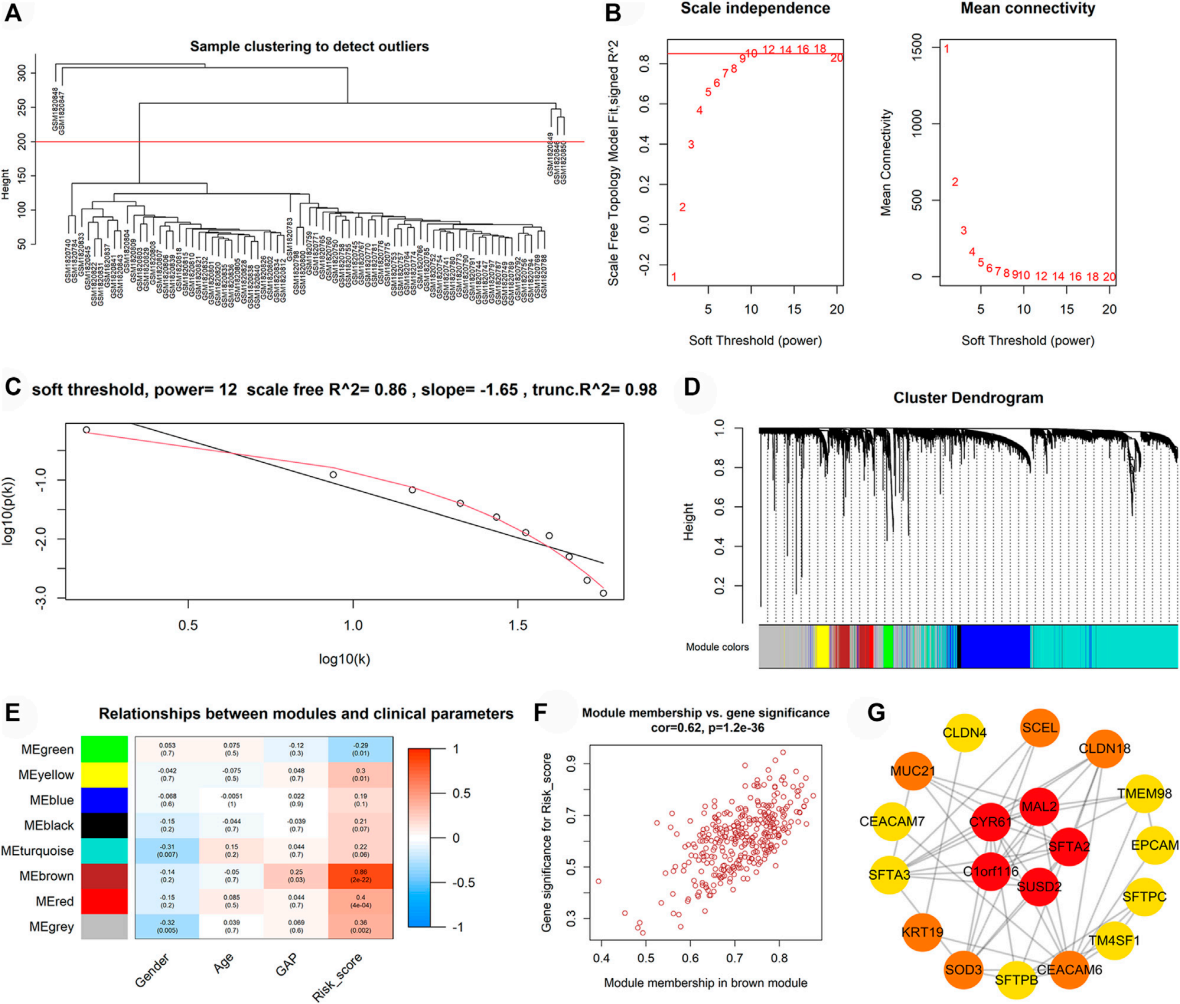
The possible hub genes of m7GPS in IPF based on the discovery cohort. **(A)** The clustering tree diagram indicates that five samples (above the red line) with distinct outliers should be excluded; the remaining 74 samples (below the red line) were utilized for subsequent analysis. **(B,C)** The soft threshold was 12 when the optimal scale-free R^2^ equaled 0.86. **(D–E)** Eight modules were determined in hierarchical clustering, of which the “brown” module had the strongest correlation with the m7GPS risk score by *Pearson* correlation coefficient. **(F)** A significant positive correlation between gene significance of risk score and module membership in the “brown” module. **(G)** Five genes (CYR61, C1ORF116, MAL2, SFTA2, and SUSD2) were hub genes of the “brown” module; the network was constructed in Cytoscape with the “degree” algorithm. In Cytoscape, the visualization network was constructed based on the top 100 genes (rank by the weight values) of the “brown” module; genes not in the most extensive network are not displayed.

For further understating the underlying mechanisms of m7GPS in IPF, the WGCNA determined candidate hub genes of m7GPS. For the discovery cohort, five samples with distinct outliers were excluded, and the remaining 74 samples were utilized for subsequent analysis (Figure 9A). With the optimal scale-free R^2^ (equaling 0.86), the soft threshold was set as 12 to construct a scale-free network (Figures 9B,C). Eight modules were determined in hierarchical clustering (Figure 9D). The “brown” module had the strongest correlation with the m7GPS risk score (*Pearson* correlation coefficient = 0.86, *p* < 0.05; Figure 9E), which was also supported by the significant positive correlation between gene significance of risk score and module membership in the “brown” module (*Pearson* correlation coefficient = 0.62, *p* < 0.05; Figure 9F). *CYR61*, *C1ORF116*, *MAL2*, *SFTA2*, and *SUSD2* were determined as the hub genes of the “brown” module in the Cytoscape (Figure 9G), indicating the five genes were essential for the m7GPS risk score in IPF based on the discovery cohort. Additionally, *CYR61* was identified as the hub gene of m7GPS risk score in the internal cohort (Supplementary Figure S3), while hub genes in the external cohort were *PPP1R14A* and *C11ORF9* (Supplementary Figure S4).

**FIGURE 10:**
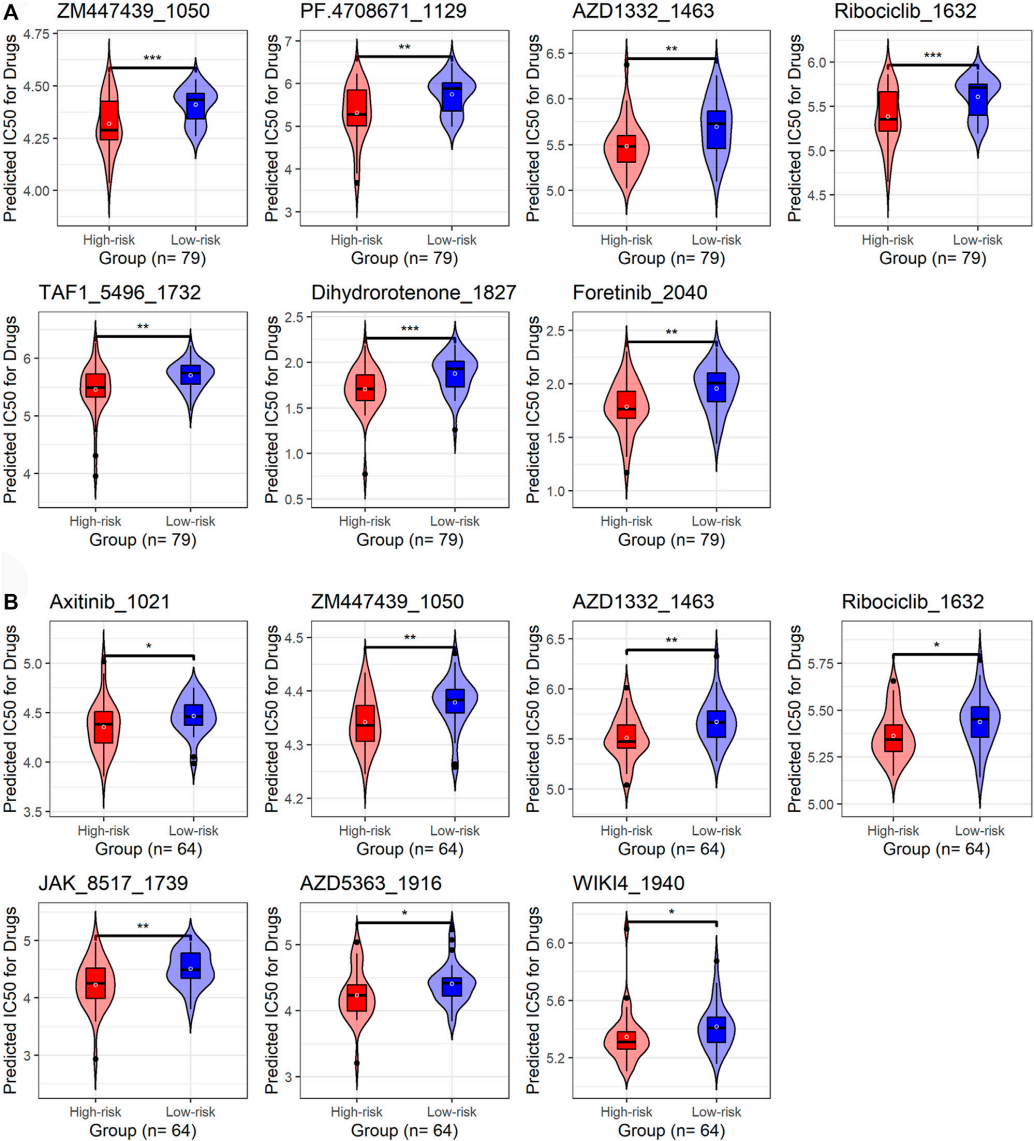
The underlying target drugs for m7GPS based on half-limiting dose values. **(A)** Discovery cohort. **(B)** External validation cohort. The high-risk and low-risk groups are classified based on the median risk score of the cohort; *p*-values were calculated based on Wilcoxon rank-sum tests; *p < 0.05; **p < 0.02; ***p < 0.001.

Using the “oncoPredict” package, this study predicted drugs potentially applicable to IPF patients with high risk scores. In this research, seven drugs that may be related to m7GPS were obtained from the discovery and validation cohorts based on the bilateral *p*-value < 0.01. Furthermore, the unilateral *p* values of these seven drugs were calculated to select drugs sensitive to high-risk IPF patients. As a result, in both the discovery and validation cohorts, ZM447439-1050, AZD1332-1463, and Ribociclib-1632 were identified as drugs that might apply to patients with high-risk scores (Figures 10A,B).

## 4 Discussion

Among interstitial pulmonary diseases, IPF has the highest incidence and mortality. Due to the lack of specific markers, it is difficult for clinicians to accurately evaluate the prognosis of IPF patients, increasing the difficulty of the diagnosis and treatment of patients. Prognostic models are effective indexes for assessing the prognosis of patients and have been widely concerned in quite a few diseases, especially in tumors. However, limited reports on prognosis models of non-neoplastic diseases, including IPF, can be consulted.

This study explores a novel potential clinical marker suitable for IPF by constructing a prognostic signature. We focused on the role of m7GRGs in IPF and constructed a novel m7GPS consisting of six genes. The m7GPS made it feasible to predict 1-, 3-, and 5-year survival probabilities of IPF patients, and it was an independent prognostic risk factor. Moreover, the underlying clinical value of m7GPS was also revealed by identifying the ability of m7GPS to screen IPF. Last, this study also explored the potential molecular mechanisms and drugs of IPF from the perspective of m7G for the first time, which promoted the understanding of IPF to some extent.

The two m7GRGs (*METTL1* and *RNMT*) and the subtypes based on them represented essential roles in IPF. According to previous reports, Dai et al. (2021) identified that *METTL1* overexpression was associated with poor prognosis in patients with intrahepatic cholangiocarcinoma, and they also found the carcinogenic role of *METTL1*-mediated m7G tRNA modification in promoting intrahepatic cholangiocarcinoma. Ying et al. (2021) also reported that *METTL1*-mediated m7G tRNA modification regulated the expression of target genes such as *EGFR* and *EFEMP1*, thereby accelerating bladder cancer progression. Our study identified upregulated *METTL1* in BLA cells of IPF patients, and increasing levels of *METTL1* indicated a poor prognosis of IPF; to our knowledge, the prognostic value of overexpressed *METTL1* in IPF has not been reported before, indicating the novelty of this finding. The other m7GRG, *RNMT*, was considered to promote the proliferation of tumor cells by Chen et al. (2020). However, our study did not find the statistical difference in the expression of *RNMT* in BLA cells of IPF patients and healthy persons and the significant prognostic value of this gene in IPF. Even so, the combination of *METTL1* and *RNMT* expression levels can classify IPF into two molecular subtypes (subtype 1 and subtype 2). The prognosis of patients in subtype 2 was more unfavorable than those in subtype 1, indicating the importance of m7G subtypes.

The m7GPS’ essential value in predicting the prognosis of IPF patients and screening IPF patients was detected in this study. The m7GPS was composed of *CCL2*, *CCL7*, *HS3ST1*, *MRVI1*, *TM4SF1*, and *TPST1*. Some reports have revealed the roles of some genes in these six genes in IPF. For example, *CCL2* enhances the ability of the body to recruit fibrogenic macrophages by participating in a particular signaling pathway, thereby promoting IPF (Tian et al., 2021). High expression of *CCL7* can be detected in biopsy samples and cell lines of patients with interstitial pneumonia, and this chemokine plays a vital role in the progression of fibrosis (Choi et al., 2004). There were no studies on *HS3ST1*, *MRVI1*, *TM4SF1*, and *TPST* in IPF. Our study highlighted the association of the four genes with poor prognosis of IPF patients according to univariate Cox regression analysis results. Indeed, three prognosis signatures have been established by the recent studies of Prasse et al. (2019), Li et al. (2021), He et al. (2022). All of their prognosis signatures could be used to predict the prognosis of IPF patients. In our study, we constructed the m7GPS from the m7G perspective for the first time. m7GPS was an independent risk factor for the prognosis of IPF patients. The accuracy of m7GPS in predicting the 1-, 3-, and 5-year survival status of IPF patients was higher than that of the ferroptosis-related signatures constructed by Li et al. (2021), He et al. (2022). Moreover, compared to the signature identified by Prasse et al. (2019), the concordance indexes (0.76–0.80) of GAP stages combined with m7GPS were slighter higher than that (0.71–0.78) of GAP stages combined with the signature of Prasse et al. (2019), which suggested the advantage of m7GPS to some extent. Furthermore, the nomogram established in our study revealed the critical potential clinical significance of m7GPS by accurately predicting the short-term survival of IPF patients. Additionally, we confirmed that m7GPS and its constituent genes were reliable indicators for screening IPF patients from healthy individuals, while similar investigations were not discussed by the prognostic signatures of Prasse et al. (2019), Li et al. (2021), He et al. (2022). In conclusion, the m7GPS constructed in our study may serve as a novel biomarker for predicting the prognosis of IPF patients and screening IPF patients.

The roles of m7GPS in IPF may link to inflammation and immune response. Heukels et al. (2019) previously emphasized the importance of inflammatory response and immune response in IPF, and the two perspectives may be essential for m7GPS in IPF with the fact that: 1) Thirteen types of molecular functions were clustered in DENGs between high- and low-risk IPF groups, including cytokine activity and chemokine activity and other categories related to inflammatory response; and 2) From the perspective of KEGG signaling pathway, viral protein interaction with cytokine and cytokine receptor was found in all of the discovery, internal, and external validation cohorts. Multiple types of immune cells were involved in the progress of IPF, and macrophage was one of them (Shenderov et al., 2021). Previous studies have shown that macrophages tended to be detected in fibrotic lung tissues rather than normal lung tissues. Derived from M0 macrophages, M1 or M2 macrophages were considered to trigger the activation of fibroblasts by producing cytokines such as TGF-β, which can promote fibrosis (Shenderov et al., 2021). In this study, although the high risk score of m7GPS was not associated with a higher level of activated M1 or M2 macrophages, a lower level of M0 macrophages was detected in IPF patients with a high risk score, which may be because part of M0 macrophages was converted into M1 or M2 macrophages and participated in the fibrosis process. Salonen et al. (2021) detected a higher density of mast cells in IPF tissue than in the control lung tissue. Further, Shimbori et al. (2019) revealed that the increasing mast cells were associated with the severity of IPF. Attractively, high risk scores were positively associated with increasing levels of activated mast cells, suggesting that the risk factor of m7GPS for poor prognosis in IPF may link to the increased level of activated mast cells, while this needs further verification in future experiments.

Several genes—*CYR61*, *C1ORF116*, *MAL2*, *SFTA2*, *SUSD2*, *PPP1R14A*, and *C11ORF9*—were determined as the hub genes of m7GPS in IPF. Among these genes, Grazioli et al. (2015) found that *CYR61* was overexpressed in the lung injury area of IPF patients and speculated that the gene enhanced lung injury. However, no research about the other hub genes on IPF has been discussed, which requires more exploration. Additionally, taking into account the limited clinical use of pirfenidone and nintedanib for the treatment of IPF patients with different characteristics (King et al., 2014b; Richeldi et al., 2014; Raghu et al., 2015), we explored and demonstrated three drugs (ZM447439-1050, AZD1332-1463, and Ribociclib-1632) that were sensitive to patients with high m7GPS risk scores, which may provide implications for subsequent IPF-related drug research.

There are several limitations to this study. The sample size used to construct and validate m7GPS is small, which may be why the nomogram has relatively low accuracy in predicting long-term survival in IPF patients. At the same time, sufficient clinical parameter data were not collected in this study to further explore the clinical correlation of m7GPS. In the future, it is necessary to collect in-house clinical data for experimental verification of some findings in this study, such as the potential drugs sensitive to IPF patients with high m7GPS risk scores and the corresponding mechanisms of these drugs on IPF. Additionally, the current study established the m7GPS based on only LASSO Cox regression; the prognostic signature established by other methods (at least Random Forest) is also worthy of further investigation.

Collectively, this study constructed a novel m7GPS composed of six genes. m7GPS is a reliable indicator for predicting the survival probability of IPF patients and is identified as an effective marker for prognosis and screening of IPF patients. The underlying mechanisms of m7GPS in IPF may relate to inflammation and immune response. Several drugs potentially sensitive to patients with high m7GPS risk scores were also investigated. In a word, m7GPS may serve as a novel marker for IPF.

## Data Availability

Publicly available datasets were analyzed in this study. The names of the repository/repositories and accession number(s) can be found in the article/Supplementary Material.
