# Supplementary material for: Construction and Validation of a Novel Prognostic Signature of Idiopathic Pulmonary Fibrosis by Identifying Subtypes Based on Genes Related to 7-Methylguanosine Modification
Source: Front Genet. 2022 Jun 9;13:890530. doi: 10.3389/fgene.2022.890530 (PMC9218869; doi:10.3389/fgene.2022.890530)
Supplement: Supplementary file 1 [file Table1.DOCX]

**Supplementary Table S1.** The m7G subtypes condition of the Freiburg-Siena cohort.

| IPF patients | Subtype |  | IPF patients | Subtype |  | IPF patients | Subtype |
| --- | --- | --- | --- | --- | --- | --- | --- |
| GSM1820739 | 1 |  | GSM1820764 | 2 |  | GSM1820809 | 2 |
| GSM1820740 | 1 |  | GSM1820765 | 2 |  | GSM1820810 | 2 |
| GSM1820741 | 1 |  | GSM1820766 | 1 |  | GSM1820811 | 2 |
| GSM1820742 | 1 |  | GSM1820768 | 2 |  | GSM1820812 | 2 |
| GSM1820745 | 1 |  | GSM1820769 | 2 |  | GSM1820813 | 2 |
| GSM1820750 | 1 |  | GSM1820771 | 1 |  | GSM1820814 | 2 |
| GSM1820752 | 1 |  | GSM1820772 | 1 |  | GSM1820815 | 2 |
| GSM1820753 | 1 |  | GSM1820773 | 2 |  | GSM1820816 | 2 |
| GSM1820754 | 1 |  | GSM1820775 | 2 |  | GSM1820817 | 2 |
| GSM1820755 | 1 |  | GSM1820776 | 1 |  | GSM1820818 | 2 |
| GSM1820757 | 1 |  | GSM1820777 | 2 |  | GSM1820819 | 2 |
| GSM1820758 | 1 |  | GSM1820778 | 1 |  | GSM1820820 | 2 |
| GSM1820759 | 1 |  | GSM1820779 | 1 |  | GSM1820821 | 2 |
| GSM1820760 | 1 |  | GSM1820781 | 1 |  | GSM1820822 | 2 |
| GSM1820761 | 1 |  | GSM1820783 | 2 |  | GSM1820823 | 2 |
| GSM1820763 | 1 |  | GSM1820785 | 1 |  | GSM1820825 | 2 |
| GSM1820767 | 1 |  | GSM1820786 | 2 |  | GSM1820826 | 1 |
| GSM1820770 | 1 |  | GSM1820787 | 2 |  | GSM1820827 | 1 |
| GSM1820774 | 1 |  | GSM1820788 | 2 |  | GSM1820828 | 2 |
| GSM1820780 | 1 |  | GSM1820789 | 2 |  | GSM1820829 | 2 |
| GSM1820782 | 1 |  | GSM1820791 | 1 |  | GSM1820830 | 2 |
| GSM1820784 | 1 |  | GSM1820792 | 2 |  | GSM1820831 | 2 |
| GSM1820790 | 1 |  | GSM1820793 | 2 |  | GSM1820832 | 2 |
| GSM1820824 | 2 |  | GSM1820794 | 2 |  | GSM1820833 | 2 |
| GSM1820845 | 1 |  | GSM1820795 | 2 |  | GSM1820834 | 2 |
| GSM1820846 | 2 |  | GSM1820796 | 1 |  | GSM1820835 | 2 |
| GSM1820847 | 2 |  | GSM1820797 | 2 |  | GSM1820836 | 2 |
| GSM1820849 | 2 |  | GSM1820798 | 2 |  | GSM1820837 | 2 |
| GSM1820850 | 2 |  | GSM1820799 | 2 |  | GSM1820838 | 2 |
| GSM1820743 | 1 |  | GSM1820800 | 2 |  | GSM1820839 | 2 |
| GSM1820744 | 1 |  | GSM1820801 | 2 |  | GSM1820840 | 2 |
| GSM1820746 | 1 |  | GSM1820802 | 2 |  | GSM1820841 | 2 |
| GSM1820747 | 2 |  | GSM1820803 | 2 |  | GSM1820842 | 2 |
| GSM1820748 | 2 |  | GSM1820804 | 2 |  | GSM1820843 | 2 |
| GSM1820749 | 2 |  | GSM1820805 | 1 |  | GSM1820844 | 2 |
| GSM1820751 | 1 |  | GSM1820806 | 2 |  | GSM1820848 | 2 |
| GSM1820756 | 2 |  | GSM1820807 | 2 |  |  |  |
| GSM1820762 | 2 |  | GSM1820808 | 1 |  |  |  |
