# Supplementary figures and images for: Construction and Validation of a Novel Prognostic Signature of Idiopathic Pulmonary Fibrosis by Identifying Subtypes Based on Genes Related to 7-Methylguanosine Modification
Source: Front Genet. 2022 Jun 9;13:890530. doi: 10.3389/fgene.2022.890530 (PMC9218869; doi:10.3389/fgene.2022.890530)

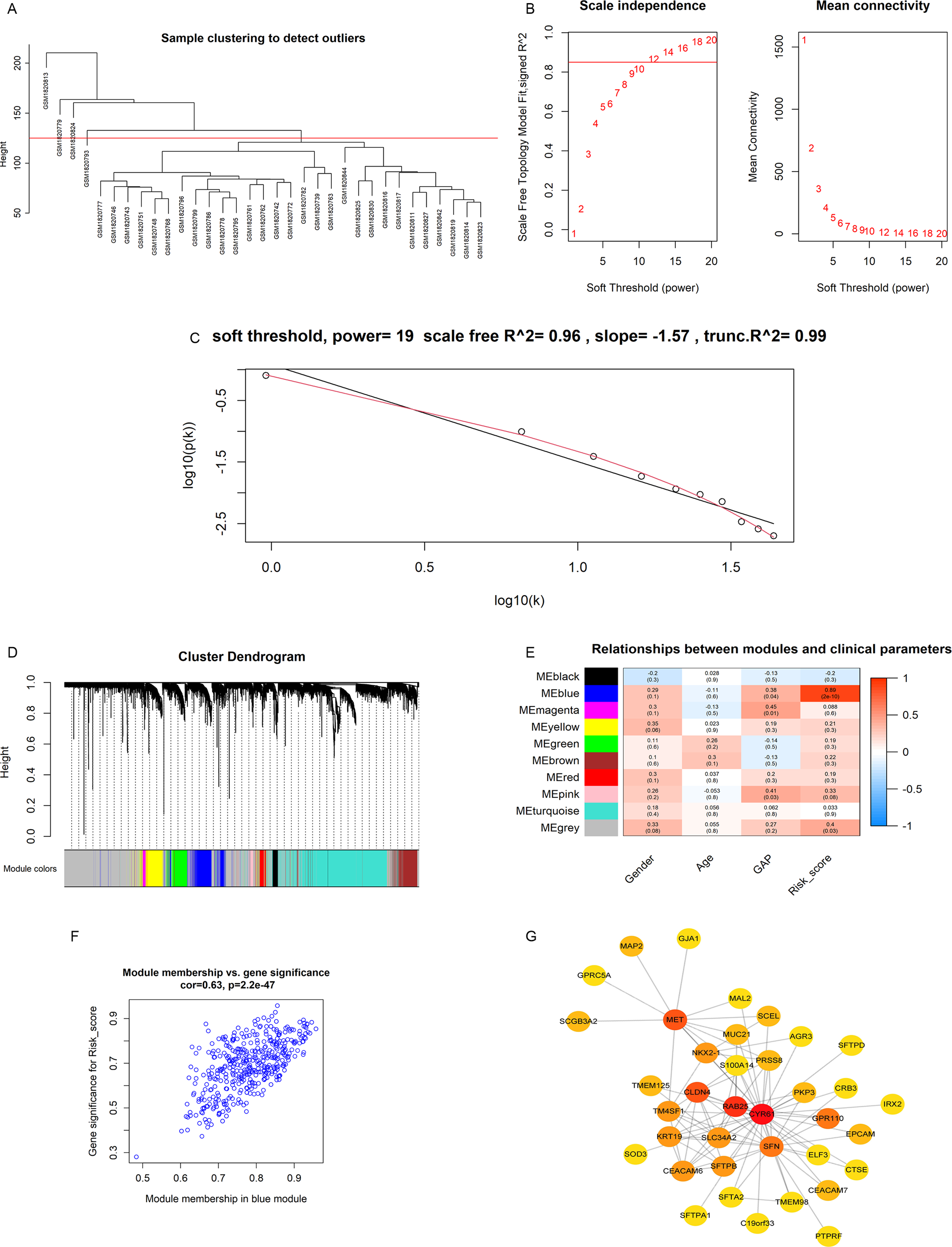

Supplement: Supplementary file 2 [file Image3.TIF]

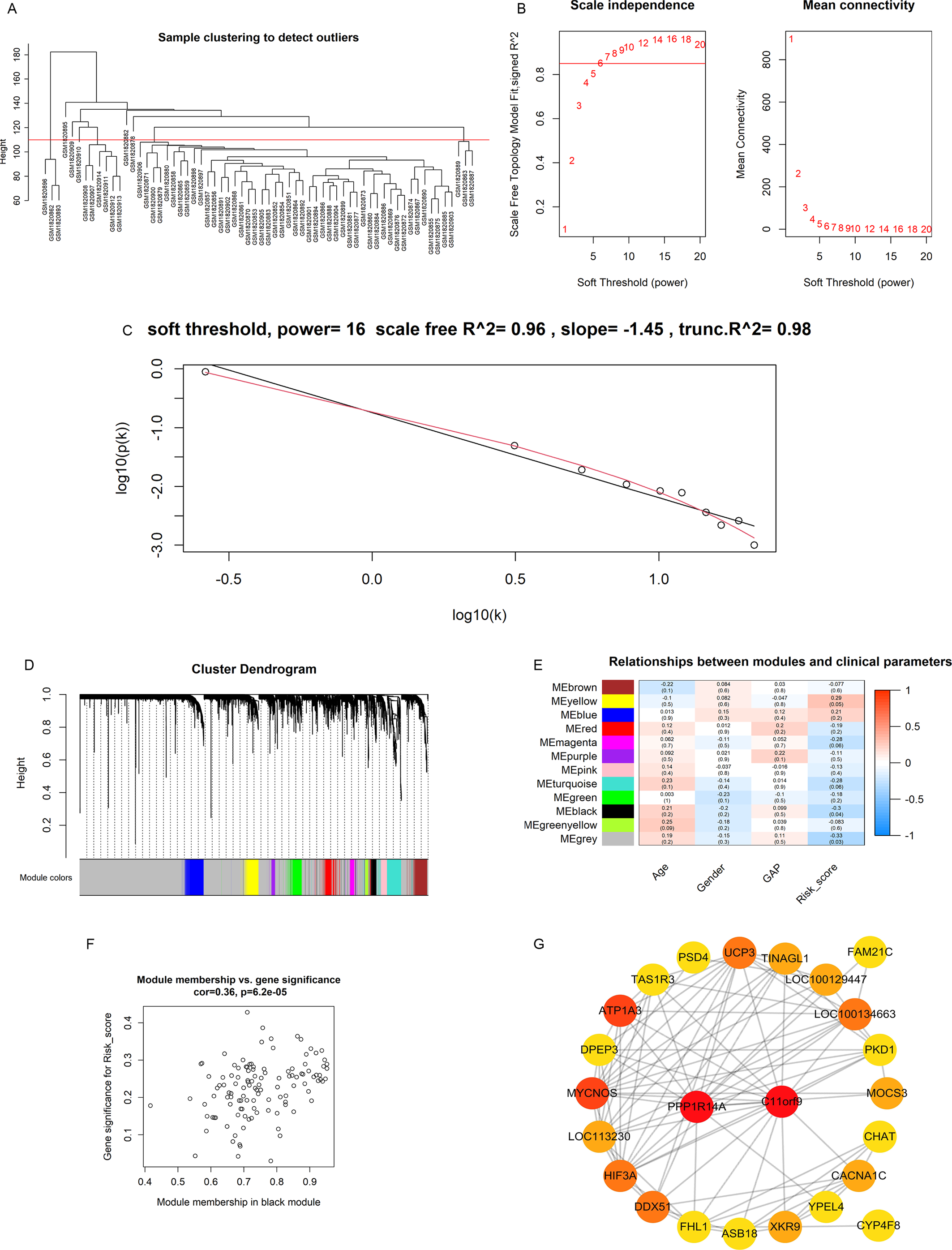

Supplement: Supplementary file 3 [file Image4.TIF]

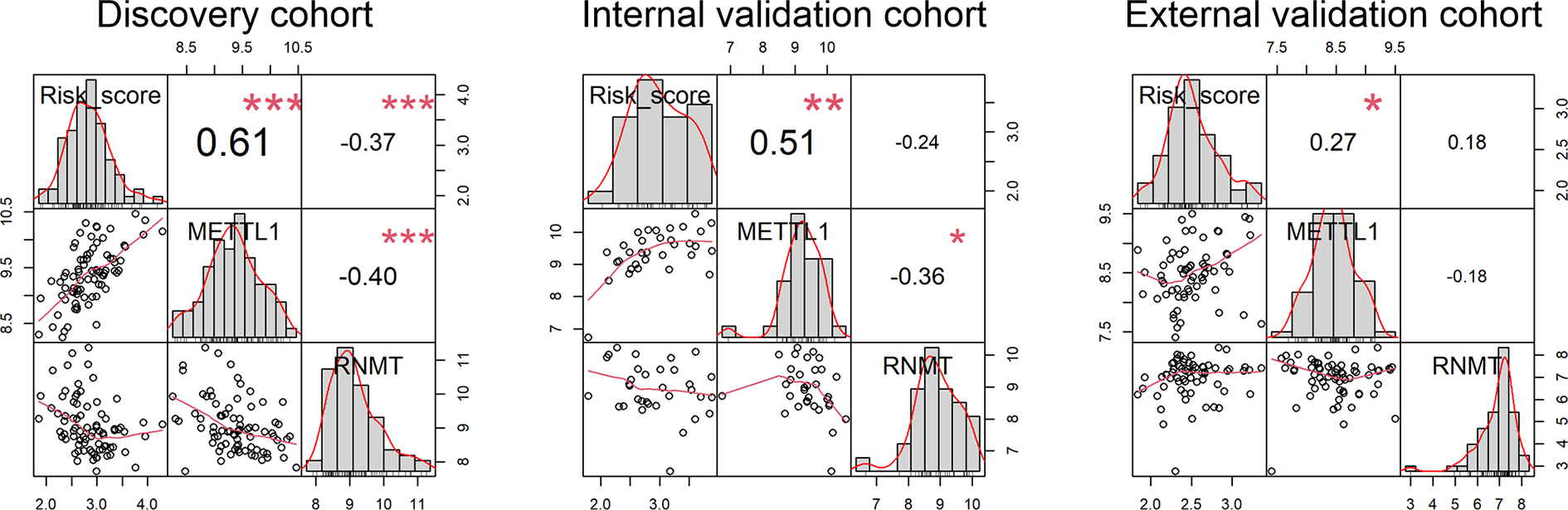

Supplement: Supplementary file 4 [file Image2.TIF]

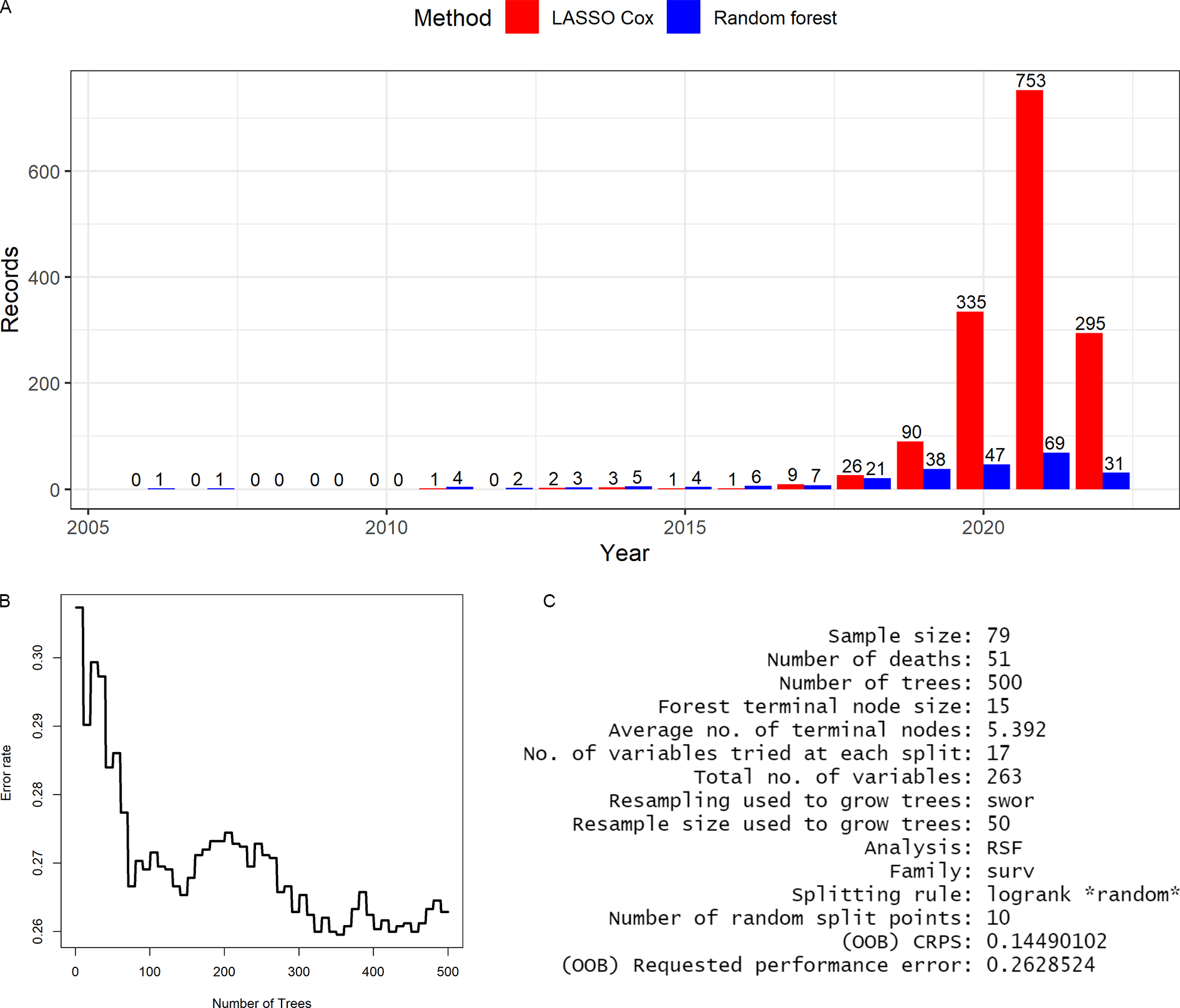

Supplement: Supplementary file 5 [file Image1.TIF]
