# Supplementary material for: Construction and Validation of a Novel Prognostic Signature of Idiopathic Pulmonary Fibrosis by Identifying Subtypes Based on Genes Related to 7-Methylguanosine Modification
Source: Front Genet. 2022 Jun 9;13:890530. doi: 10.3389/fgene.2022.890530 (PMC9218869; doi:10.3389/fgene.2022.890530)
Supplement: Supplementary file 6 [file Table2.DOCX]

**Supplementary Table S2.** The discovery cohort in this study.

| ID of IPF patient | Survival  time | Status | Gender | Age | GAP | CCL2 | CCL7 | HS3ST1 | MRVI1 | TM4SF1 | TPST1 | Risk  score |
| --- | --- | --- | --- | --- | --- | --- | --- | --- | --- | --- | --- | --- |
| 1820740 | 0.836 | Dead | Male | 76 | 5 | 10.78 | 5.268 | 6.905 | 7.943 | 6.06 | 5.756 | 2.605 |
| 1820741 | 1.526 | Dead | Male | 68 | 6 | 11.5 | 7.089 | 6.493 | 6.183 | 7.012 | 2.913 | 2.575 |
| 1820745 | 0.458 | Dead | Male | 54 | 3 | 11.92 | 7.006 | 7.872 | 4.25 | 5.343 | 3.437 | 2.628 |
| 1820750 | 2.69 | Dead | Male | 58 | 1 | 10.5 | 5.962 | 6.096 | 5.342 | 2.537 | 3.105 | 2.127 |
| 1820752 | 5.896 | Alive | Male | 76 | 5 | 9.563 | 3.794 | 6.386 | 2.572 | 4.502 | 2.964 | 2.099 |
| 1820753 | 2.222 | Dead | Male | 75 | 5 | 11.72 | 5.787 | 7.637 | 6.015 | 4.654 | 3.855 | 2.598 |
| 1820754 | 6.844 | Alive | Male | 74 | 4 | 10.16 | 5.553 | 6.153 | 5.018 | 4.593 | 3.505 | 2.226 |
| 1820755 | 0.266 | Dead | Male | 76 | 8 | 12.02 | 6.63 | 9.299 | 4.044 | 5.597 | 3.619 | 2.829 |
| 1820757 | 2.258 | Dead | Male | 76 | 7 | 10.85 | 6.066 | 7.603 | 4.988 | 5.598 | 3.606 | 2.538 |
| 1820758 | 1.699 | Dead | Female | 72 | 4 | 11.7 | 6.991 | 7.145 | 3.188 | 5.556 | 4.45 | 2.513 |
| 1820759 | 5.384 | Alive | Male | 56 | 2 | 11.25 | 6.851 | 7.206 | 2.561 | 5.107 | 3.63 | 2.416 |
| 1820760 | 6.129 | Alive | Male | 63 | 5 | 10.28 | 4.628 | 6.906 | 4.628 | 4.54 | 3.614 | 2.314 |
| 1820767 | 4.041 | Dead | Male | 53 | 2 | 12.48 | 6.615 | 8.165 | 5.283 | 6.612 | 3.276 | 2.824 |
| 1820770 | 3.123 | Dead | Male | 75 | 4 | 12.06 | 7.834 | 9.063 | 4.098 | 7.514 | 3.286 | 2.924 |
| 1820774 | 2.447 | Dead | Male | 54 | 2 | 12.96 | 8.395 | 8.168 | 6.978 | 8.376 | 6.132 | 3.093 |
| 1820780 | 2.66 | Alive | Male | 68 | 3 | 10.5 | 4.779 | 7.658 | 2.556 | 4.855 | 3.022 | 2.37 |
| 1820784 | 0.575 | Dead | Male | 59 | 5 | 14.39 | 9.596 | 8.002 | 3.767 | 6.455 | 7.311 | 2.998 |
| 1820790 | 1.679 | Alive | Male | 69 | 3 | 9.925 | 6.269 | 7.459 | 3.607 | 5.226 | 3 | 2.358 |
| 1820845 | 1.258 | Alive | Male | 60 | 3 | 10.71 | 6.252 | 7.375 | 5.799 | 6.092 | 4.059 | 2.563 |
| 1820744 | 1.967 | Dead | Male | 77 | 5 | 10.83 | 6.301 | 7.966 | 4.802 | 5.852 | 4.157 | 2.603 |
| 1820766 | 2.671 | Dead | Male | 63 | 5 | 11.3 | 6.496 | 7.928 | 6.847 | 4.676 | 6.355 | 2.674 |
| 1820771 | 4.967 | Alive | Male | 72 | 4 | 8.242 | 4.119 | 6.486 | 2.583 | 2.964 | 2.235 | 1.886 |
| 1820776 | 4.003 | Alive | Male | 74 | 5 | 6.994 | 2.354 | 7.065 | 3.242 | 2.401 | 3.192 | 1.845 |
| 1820781 | 1.641 | Dead | Male | 67 | 6 | 14.18 | 9.98 | 9.137 | 7.028 | 8.811 | 7.943 | 3.393 |
| 1820785 | 1.912 | Alive | Male | 54 | 4 | 12.5 | 7.95 | 7.329 | 5.389 | 6.47 | 7.883 | 2.803 |
| 1820791 | 1.627 | Alive | Male | 71 | 3 | 10.47 | 6.344 | 7.471 | 3.524 | 4.777 | 2.937 | 2.377 |
| 1820805 | 2.301 | Dead | Female | 54 | 1 | 10.3 | 5.717 | 7.801 | 4.789 | 6.098 | 2.891 | 2.525 |
| 1820808 | 2.389 | Alive | Female | 68 | 6 | 12.67 | 8.228 | 6.855 | 5.833 | 6.089 | 3.926 | 2.679 |
| 1820826 | 0.871 | Dead | Male | 73 | 8 | 13.71 | 8.446 | 8.948 | 5.828 | 7.373 | 6.936 | 3.172 |
| 1820846 | 1.167 | Dead | Male | 71 | 3 | 11.1 | 8.452 | 9.475 | 2.092 | 9.164 | 6.556 | 2.988 |
| 1820847 | 1.099 | Dead | Male | 76 | 5 | 10.79 | 9.684 | 9.101 | 11.48 | 8.014 | 5.961 | 3.147 |
| 1820849 | 1.011 | Dead | Male | 62 | 7 | 12.21 | 7.711 | 8.309 | 7.923 | 4.555 | 9.467 | 2.893 |
| 1820850 | 0.668 | Dead | Male | 55 | 4 | 13.68 | 13.18 | 10.43 | 7.884 | 11.94 | 8.364 | 3.758 |
| 1820747 | 1.573 | Dead | Male | 49 | 2 | 10.14 | 5.401 | 7.5 | 2.544 | 4.162 | 2.628 | 2.27 |
| 1820749 | 2.962 | Dead | Female | 78 | 5 | 11.99 | 7.716 | 7.679 | 5.473 | 6.197 | 5.09 | 2.736 |
| 1820756 | 0.225 | Dead | Male | 72 | 5 | 12.22 | 8.371 | 8.251 | 6.176 | 7.372 | 8.541 | 2.991 |
| 1820764 | 3.222 | Dead | Male | 65 | 6 | 13.45 | 8.341 | 8.499 | 4.829 | 6.735 | 5.349 | 2.991 |
| 1820765 | 0.605 | Dead | Male | 78 | 5 | 12.84 | 7.477 | 9.628 | 6.844 | 6.57 | 5.154 | 3.129 |
| 1820769 | 0.759 | Dead | Male | 74 | 6 | 14.18 | 9.153 | 9.399 | 7.96 | 7.27 | 7.223 | 3.344 |
| 1820773 | 2.479 | Dead | Male | 77 | 8 | 11.73 | 8.064 | 8.415 | 4.975 | 6.346 | 3.901 | 2.78 |
| 1820775 | 4.329 | Alive | Male | 68 | 3 | 11.46 | 7.715 | 8.112 | 5.909 | 4.721 | 5.032 | 2.665 |
| 1820783 | 0.162 | Dead | Male | 66 | 7 | 13.26 | 10.7 | 8.686 | 7.855 | 10.9 | 8.823 | 3.431 |
| 1820787 | 1.836 | Alive | Male | 67 | 4 | 10.68 | 6.273 | 6.532 | 3.442 | 5.929 | 3.604 | 2.358 |
| 1820788 | 0.268 | Dead | Female | 71 | 4 | 13.5 | 8.484 | 9.118 | 6.12 | 6.282 | 6.616 | 3.111 |
| 1820789 | 1.742 | Alive | Male | 84 | 4 | 11.84 | 6.792 | 8.298 | 5.108 | 5.894 | 4.008 | 2.747 |
| 1820792 | 0.838 | Dead | Male | 48 | 5 | 14.88 | 10.82 | 9.652 | 6.291 | 8.471 | 7.334 | 3.467 |
| 1820794 | 1.548 | Dead | Male | 71 | 5 | 12.48 | 8.234 | 7.741 | 5.818 | 9.093 | 8.755 | 3.048 |
| 1820797 | 2.299 | Alive | Male | 65 | 7 | 10.38 | 5.903 | 6.878 | 2.625 | 5.3 | 3.913 | 2.312 |
| 1820798 | 0.181 | Dead | Male | 44 | 6 | 16.68 | 12.38 | 10.79 | 9.224 | 8.881 | 10.21 | 3.954 |
| 1820800 | 0.058 | Dead | Male | 53 | 4 | 15.49 | 9.926 | 11.65 | 10.15 | 7.956 | 10.03 | 3.916 |
| 1820801 | 0.244 | Dead | Male | 51 | 2 | 12.41 | 7.721 | 7.233 | 6.574 | 7.492 | 7.824 | 2.884 |
| 1820802 | 1.51 | Alive | Male | 60 | 1 | 12.42 | 7.678 | 7.324 | 5.269 | 5.877 | 3.117 | 2.667 |
| 1820803 | 0.351 | Dead | Male | 49 | 2 | 13.55 | 8.293 | 9.235 | 9.058 | 7.856 | 7.536 | 3.342 |
| 1820804 | 1.282 | Alive | Male | 83 | 8 | 11.66 | 6.194 | 7.264 | 5.152 | 5.721 | 5.144 | 2.608 |
| 1820806 | 0.54 | Dead | Male | 82 | 7 | 14.29 | 10.44 | 10.5 | 6.515 | 8.651 | 7.235 | 3.539 |
| 1820807 | 1.293 | Dead | Female | 82 | 7 | 13.45 | 9.225 | 10.02 | 7.38 | 9.94 | 10.14 | 3.558 |
| 1820809 | 2.156 | Dead | Female | 81 | 3 | 13.77 | 9.289 | 9.428 | 4.356 | 4.988 | 4.172 | 2.995 |
| 1820810 | 2.888 | Alive | Male | 75 | 4 | 10.95 | 6.279 | 7.142 | 5.165 | 6.652 | 3.842 | 2.566 |
| 1820812 | 1.036 | Dead | Male | 62 | 7 | 12.7 | 7.898 | 8.792 | 4.925 | 5.453 | 3.915 | 2.856 |
| 1820815 | 2.625 | Dead | Male | 86 | 6 | 12.68 | 8.183 | 9.171 | 5.688 | 5.799 | 6.06 | 2.989 |
| 1820818 | 3.027 | Alive | Female | 81 | 3 | 10.99 | 6.148 | 6.223 | 4.425 | 5.579 | 5.005 | 2.381 |
| 1820820 | 0.301 | Dead | Male | 78 | 7 | 14.12 | 9.626 | 8.301 | 6.875 | 8.9 | 7 | 3.264 |
| 1820821 | 1.293 | Dead | Male | 64 | 5 | 14.98 | 9.79 | 8.556 | 8.231 | 6.34 | 7.409 | 3.265 |
| 1820822 | 0.381 | Dead | Male | 79 | 6 | 13.71 | 8.343 | 8.613 | 7.494 | 6.826 | 9.384 | 3.195 |
| 1820828 | 3.005 | Alive | Female | 68 | 2 | 11.39 | 6.732 | 6.566 | 5.424 | 6.347 | 4.803 | 2.54 |
| 1820829 | 0.115 | Dead | Male | 72 | 8 | 17.06 | 12.25 | 12.72 | 10.24 | 9.32 | 10.36 | 4.295 |
| 1820831 | 0.216 | Dead | Male | 62 | 7 | 13.86 | 9.135 | 8.038 | 5.883 | 7.426 | 8.94 | 3.114 |
| 1820832 | 2.814 | Alive | Male | 72 | 5 | 11.36 | 5.904 | 7.609 | 3.683 | 5.553 | 3.476 | 2.535 |
| 1820833 | 1.526 | Dead | Male | 55 | 4 | 13.83 | 8.511 | 10.23 | 7.264 | 6.342 | 6.629 | 3.323 |
| 1820834 | 1.808 | Alive | Male | 40 | 2 | 12.86 | 7.308 | 7.615 | 4.475 | 4.489 | 3.797 | 2.641 |
| 1820835 | 0.395 | Dead | Male | 72 | 7 | 11.97 | 7.192 | 8.018 | 6.101 | 7.688 | 6.973 | 2.923 |
| 1820836 | 0.742 | Dead | Male | 73 | 4 | 12.39 | 6.588 | 7.926 | 6.112 | 7.758 | 4.204 | 2.902 |
| 1820837 | 1.696 | Alive | Male | 62 | 4 | 12.3 | 8.295 | 8.184 | 6.714 | 5.063 | 5.498 | 2.808 |
| 1820838 | 2.353 | Alive | Female | 79 | 3 | 12.02 | 7.491 | 8.181 | 5.206 | 5.073 | 5.522 | 2.731 |
| 1820839 | 1.597 | Alive | Female | 88 | 3 | 14 | 7.176 | 9.185 | 6.997 | 6.468 | 4.942 | 3.17 |
| 1820840 | 2.003 | Dead | Female | 62 | 6 | 12.93 | 7.699 | 9.26 | 6.22 | 6.022 | 5.944 | 3.05 |
| 1820841 | 1.504 | Alive | Male | 41 | 3 | 11.76 | 6.428 | 7.595 | 4.301 | 5.998 | 3.999 | 2.629 |
| 1820843 | 1.479 | Dead | Male | 75 | 4 | 13.15 | 8.555 | 8.678 | 4.789 | 7.137 | 6.613 | 3.034 |
| 1820848 | 1.049 | Alive | Male | 71 | 4 | 12.06 | 9.597 | 9.478 | 4.635 | 3.436 | 14.31 | 2.943 |
