# Supplementary material for: Construction and Validation of a Novel Prognostic Signature of Idiopathic Pulmonary Fibrosis by Identifying Subtypes Based on Genes Related to 7-Methylguanosine Modification
Source: Front Genet. 2022 Jun 9;13:890530. doi: 10.3389/fgene.2022.890530 (PMC9218869; doi:10.3389/fgene.2022.890530)
Supplement: Supplementary file 7 [file DataSheet1.PDF]

FIGURE 1. The design and main results of this study. IPF, idiopathic pulmonary fibrosis; m7GPS, m7G-related prognostic signature.

FIGURE 2. The expression and prognostic significance of m7G regulatory genes (m7GRGs) in IPF. **(A)** Differences in expression of m7GRGs between IPF and normal bronchoalveolar lavage cells (synthetic minority oversampling technique data were used in this panel); the *p*-value is calculated based on *Wilcoxon* rank-sum tests. **(B)** The association of m7GRGs expression levels with the prognosis of IPF patients; the p-value is calculated based on log-rank tests. **(C)** The cumulative distribution function plot demonstrates that among 2–20, the optimal *k* value of subtypes equals 2. **(D)** Heatmaps of the consensus matrices for $k = 2$; the panel shows that patients can be significantly divided into two subtypes (42 patients in the subtype 1, and 70 patients in the subtype 2). **(E)** The different prognosis between the two subtypes; the *p*-value is calculated based on log-rank tests.

FIGURE 3. The construction of m7GPS. **(A–B)** Two hundred sixty-three genes were identified as candidate genes for m7GPS; DEGs, differentially expressed genes; PRGs, prognostic genes. **(C)** least absolute shrinkage and selection operator (LASSO) coefficient profiles of the 263 candidate genes. **(D)** The coefficient profile plot was produced against the log (lambda) sequence in the LASSO model, and the optimal parameter (lambda) was selected when the gene number equals 6. **(E)** Coefficients of six genes in m7GPS. **(F)** Differential expression levels of six genes between the healthy group (the smaller fan-shaped region) and the IPF group (the larger fan-shaped region); the “complete” clustering method of “phearmap” package was used in this panel. **(G)** Co-expression relationship of six genes in the healthy group (the top panel) and the IPF group (the bottom panel); *Spearman* correlation coefficient was used in this panel; $^{*}p < 0.05$; $^{**}p < 0.02$; $^{***}p < 0.001$.

FIGURE 4. The prediction accuracy and prognostic value of m7GPS in IPF. **(A)** The accuracy of m7GPS in predicting the one-, three-, and five-year survival status (alive or dead) of IPF patients. **(B)** The accuracy of m7GPS and single gene in predicting the 1-survival status of IPF patients. **(C)** Risk plots and heatmaps of the expression profile of m7GPS and clinical characteristics in the discovery, internal verification, and external validation cohorts. **(D)** The different prognosis between the high-risk (patients with a risk score less than the median value of the cohort) and low-risk (the other patients) groups of the discovery, internal verification, and external validation cohorts; the p-value is calculated based on log-rank tests.

FIGURE 5. The prognostic value of m7GPS and clinical characteristics in IPF. **(A)** Univariate Cox regression analysis. **(B)** Multiple Cox regression analysis; $^{***}p < 0.001$. GAP, gender-age-physiologic variables.

FIGURE 6. The accuracy of m7GPS and its constitutive genes in distinguishing IPF bronchoalveolar lavage and healthy lung bronchoalveolar lavage. AUC, area under the curve. Synthetic minority oversampling technique data were used in this figure.

FIGURE 7. Correlation between m7GPS and clinical features and an application of the m7CPS in the nomogram. **(A)** Correlation between m7GPS and clinical features; the *p*-value is calculated based on *Wilcoxon* rank-sum tests. **(B)** The nomogram is based on the GAP and m7GPS; the red dots in the nomogram represent the clinical features of patient GSM1820740. **(C)** Calibration curves assess the

accuracy of the nomogram in predicting the survival probabilities of IPF individuals. **(D)** Decision curve analysis (DCA) demonstrates positive net benefits of the nomogram for IPF patients.

FIGURE 8. The potential molecular mechanisms of m7GPS in IPF. **(A)** Selection of overlap molecular functions of m7GPS in the three cohorts. **(B)** The panel was drawn based on the validation cohort with the overlapped molecular functions of m7GPS in the three cohorts. **(C)** KEGG signaling pathways of the m7GPS are based on the three cohorts. **(D)** Selection of KEGG signaling pathways of m7GPS in the three cohorts. **(E)** The immune cell fraction levels between patients with high- and low-risk scores; *p*-value was calculated based on *Wilcoxon* rank-sum tests.

FIGURE 9. The possible hub genes of m7GPS in IPF based on the discovery cohort. **(A)** The clustering tree diagram indicates that five samples (above the red line) with distinct outliers should be excluded; the remaining 74 samples (below the red line) were utilized for subsequent analysis. **(B–C)** The soft threshold was 12 when the optimal scale-free R^2 equaled 0.86. **(D–E)** Eight modules were determined in hierarchical clustering, of which the “brown” module had the strongest correlation with the m7GPS risk score by *Pearson* correlation coefficient. **(F)** A significant positive correlation between gene significance of risk score and module membership in the “brown” module. **(G)** Five genes (CYR61, C1ORF116, MAL2, SFTA2, and SUSD2) were hub genes of the “brown” module; the network was constructed in Cytoscape with the “degree” algorithm. In Cytoscape, the visualization network was constructed based on the top 100 genes (rank by the weight values) of the “brown” module; genes not in the most extensive network are not displayed.

FIGURE 10. The underlying target drugs for m7GPS based on half-limiting dose values. **(A)** Discovery cohort. **(B)** External validation cohort. The high-risk and low-risk groups are classified based on the median risk score of the cohort; *p*-values were calculated based on *Wilcoxon* rank-sum tests; $^{*}p < 0.05$; $^{**}p < 0.02$; $^{***}p < 0.001$.
