# Supplementary material for: Construction and Validation of a Novel Prognostic Signature of Idiopathic Pulmonary Fibrosis by Identifying Subtypes Based on Genes Related to 7-Methylguanosine Modification
Source: Front Genet. 2022 Jun 9;13:890530. doi: 10.3389/fgene.2022.890530 (PMC9218869; doi:10.3389/fgene.2022.890530)
Supplement: Supplementary file 8 [file Table5.DOCX]

**Supplementary Table S5.** The external validation cohort in this study.

| ID of IPF patient | Survival  time | Status | Gender | Age | GAP | CCL2 | CCL7 | HS3ST1 | MRVI1 | TM4SF1 | TPST1 | Risk  score |
| --- | --- | --- | --- | --- | --- | --- | --- | --- | --- | --- | --- | --- |
| 1820851 | 3.953 | Dead | Female | 74 | 3 | 10.45 | 5.007 | 6.689 | 4.871 | 3.732 | 3.661 | 2.262 |
| 1820852 | 1.608 | Alive | Male | 64 | 2 | 11.26 | 6.525 | 6.848 | 4.462 | 6.052 | 3.734 | 2.494 |
| 1820853 | 0.586 | Dead | Male | 64 | 5 | 12.37 | 6.692 | 8.785 | 3.673 | 6.997 | 5.161 | 2.898 |
| 1820854 | 0.992 | Alive | Male | 58 | 4 | 12.09 | 7.763 | 7.844 | 4.112 | 5.245 | 3.578 | 2.635 |
| 1820855 | 1.282 | Dead | Male | 67 | 6 | 12.14 | 6.744 | 6.918 | 4.442 | 7.093 | 5.395 | 2.676 |
| 1820856 | 1.852 | Dead | Male | 63 | 4 | 10.67 | 5.931 | 8.334 | 5.3 | 4.363 | 4.593 | 2.565 |
| 1820857 | 3.896 | Alive | Male | 60 | 3 | 10.22 | 5.558 | 6.905 | 3.42 | 5.762 | 2.267 | 2.326 |
| 1820858 | 4.033 | Dead | Male | 64 | 4 | 10.75 | 5.331 | 6.425 | 2.696 | 6.407 | 7.53 | 2.422 |
| 1820859 | 1.186 | Alive | Male | 71 | 4 | 11.02 | 6.159 | 7.246 | 3.406 | 5.399 | 4.916 | 2.467 |
| 1820860 | 3.482 | Alive | Female | 76 | 3 | 9.433 | 2.882 | 5.712 | 2.165 | 3.157 | 2.952 | 1.9 |
| 1820861 | 3.51 | Dead | Male | 66 | 4 | 12.41 | 8.633 | 7.778 | 5.083 | 3.911 | 4.548 | 2.625 |
| 1820862 | 1.915 | Alive | Male | 65 | 3 | 9.322 | 4.003 | 5.839 | 2.463 | 6.508 | 4.219 | 2.153 |
| 1820863 | 0.962 | Dead | Male | 68 | 6 | 13 | 7.765 | 10.02 | 4.108 | 8.153 | 6.003 | 3.217 |
| 1820864 | 0.899 | Alive | Male | 77 | 4 | 12.45 | 6.973 | 7.902 | 7.233 | 5.417 | 5.685 | 2.822 |
| 1820865 | 1.449 | Alive | Male | 53 | 2 | 9.694 | 4.981 | 6.489 | 5.246 | 3.683 | 4.35 | 2.191 |
| 1820866 | 2.318 | Alive | Male | 64 | 4 | 10.58 | 5.043 | 6.554 | 2.431 | 4.693 | 3.838 | 2.239 |
| 1820867 | 1.915 | Alive | Male | 75 | 4 | 10.76 | 6.395 | 6.862 | 5.551 | 5.847 | 3.195 | 2.464 |
| 1820868 | 1.148 | Alive | Female | 62 | 2 | 11.28 | 6.468 | 7.347 | 2.821 | 3.289 | 3.259 | 2.323 |
| 1820869 | 3.433 | Alive | Male | 52 | 2 | 9.548 | 3.124 | 6.563 | 3.408 | 3.698 | 2.213 | 2.081 |
| 1820870 | 1.91 | Alive | Male | 72 | 3 | 10.66 | 5.94 | 4.922 | 2.799 | 6.114 | 3.443 | 2.137 |
| 1820871 | 3.397 | Alive | Female | 72 | 2 | 11.74 | 6.598 | 6.784 | 4.047 | 4.745 | 1.924 | 2.401 |
| 1820872 | 3.819 | Dead | Male | 71 | 7 | 13.2 | 8.213 | 6.918 | 3.624 | 5.889 | 4.473 | 2.658 |
| 1820873 | 3.942 | Alive | Male | 51 | 4 | 10.1 | 4.641 | 7.964 | 2.445 | 5.396 | 3.184 | 2.406 |
| 1820874 | 1.745 | Dead | Male | 60 | 4 | 10.24 | 5.728 | 7.298 | 2.24 | 5.006 | 5.099 | 2.342 |
| 1820875 | 2.515 | Alive | Male | 66 | 4 | 11.73 | 4.337 | 6.894 | 5.374 | 4.415 | 5.255 | 2.487 |
| 1820876 | 4.978 | Alive | Male | 74 | 6 | 11.31 | 5.451 | 5.561 | 2.149 | 5.786 | 5.325 | 2.266 |
| 1820877 | 0.819 | Dead | Male | 64 | 4 | 13.02 | 8.243 | 8.173 | 6.284 | 6.411 | 5.493 | 2.941 |
| 1820878 | 1.033 | Alive | Male | 69 | 4 | 13.74 | 6.938 | 9.746 | 4.096 | 6.728 | 5.207 | 3.141 |
| 1820879 | 1.496 | Alive | Female | 80 | 3 | 12.29 | 7.047 | 7.544 | 2.298 | 4.219 | 4.536 | 2.504 |
| 1820880 | 2.077 | Alive | Male | 74 | 4 | 10.87 | 6.057 | 6.746 | 2.141 | 6.838 | 3.454 | 2.412 |
| 1820881 | 1.482 | Dead | Male | 73 | 4 | 10.46 | 5.64 | 6.725 | 3.001 | 6.214 | 4.116 | 2.372 |
| 1820882 | 0.255 | Dead | Male | 75 | 4 | 13.34 | 7.847 | 10.16 | 4.896 | 9.049 | 6.584 | 3.358 |
| 1820883 | 2.471 | Alive | Female | 69 | 3 | 11.35 | 7.927 | 8.429 | 4.526 | 4.617 | 5.076 | 2.645 |
| 1820884 | 1.082 | Dead | Male | 78 | 5 | 9.902 | 4.199 | 6.769 | 4.394 | 4.767 | 4.289 | 2.28 |
| 1820885 | 1.822 | Alive | Male | 87 | 6 | 10.08 | 4.508 | 7.583 | 2.993 | 6.183 | 5.086 | 2.457 |
| 1820886 | 3.414 | Alive | Female | 61 | 3 | 10.22 | 5.194 | 5.161 | 2.252 | 3.324 | 3.138 | 1.926 |
| 1820887 | 0.603 | Dead | Female | 79 | 4 | 12.55 | 7.493 | 8.027 | 5.912 | 7.618 | 6.729 | 2.963 |
| 1820888 | 0.586 | Dead | Male | 71 | 4 | 10.98 | 5.807 | 7.562 | 2.228 | 5.365 | 2.364 | 2.415 |
| 1820889 | 0.953 | Alive | Male | 64 | 2 | 10.31 | 6.078 | 7.744 | 2.154 | 2.773 | 5.965 | 2.279 |
| 1820890 | 0.548 | Dead | Male | 52 | 3 | 10.68 | 5.922 | 6.986 | 2.328 | 5.397 | 4.648 | 2.362 |
| 1820891 | 0.693 | Dead | Male | 60 | 4 | 11.35 | 5.772 | 7.412 | 4.835 | 6.491 | 5.239 | 2.637 |
| 1820892 | 2.26 | Alive | Male | 62 | 2 | 13.2 | 8.586 | 8.184 | 3.621 | 5.503 | 4.293 | 2.793 |
| 1820893 | 1.066 | Alive | Female | 77 | 3 | 10.21 | 4.371 | 5.827 | 4.161 | 6.455 | 5.052 | 2.3 |
| 1820894 | 1.014 | Alive | Male | 73 | 3 | 11.21 | 5.828 | 7.796 | 3.785 | 4.752 | 4.58 | 2.518 |
| 1820895 | 1.532 | Alive | Female | 65 | 1 | 10.36 | 1.95 | 4.96 | 3.385 | 1.983 | 2.226 | 1.837 |
| 1820896 | 1.573 | Alive | Male | 76 | 4 | 12.28 | 6.149 | 7.674 | 3.936 | 8.156 | 5.824 | 2.84 |
| 1820897 | 3.849 | Alive | Male | 51 | 2 | 10.27 | 6.279 | 6.995 | 5.144 | 6.681 | 5.364 | 2.515 |
| 1820898 | 0.814 | Dead | Male | 78 | 5 | 12.93 | 7.648 | 8.367 | 6.426 | 5.822 | 5.812 | 2.928 |
| 1820899 | 0.666 | Alive | Female | 53 | 0 | 10.85 | 6.017 | 6.862 | 4.312 | 5.57 | 3.957 | 2.426 |
| 1820900 | 0.652 | Alive | Male | 80 | 5 | 9.261 | 3.682 | 6.235 | 2.218 | 5.724 | 3.459 | 2.126 |
| 1820901 | 0.649 | Alive | Male | 69 | 5 | 10.06 | 4.707 | 6.477 | 3.286 | 4.541 | 3.107 | 2.188 |
| 1820902 | 0.838 | Dead | Male | 77 | 6 | 12.03 | 7.014 | 7.974 | 3.81 | 6.192 | 4.456 | 2.708 |
| 1820903 | 2.844 | Dead | Male | 69 | 6 | 11.39 | 5.839 | 6.855 | 2.869 | 6.39 | 4.867 | 2.492 |
| 1820904 | 2.452 | Alive | Female | 69 | 2 | 10.6 | 5.483 | 6.968 | 2.292 | 5.823 | 3.227 | 2.35 |
| 1820905 | 4.912 | Alive | Male | 63 | 3 | 10.36 | 5.089 | 6.654 | 3.379 | 5.886 | 3.06 | 2.325 |
| 1820906 | 1.052 | Alive | Male | 65 | 3 | 10.74 | 7.783 | 7.719 | 3.967 | 5.917 | 4.159 | 2.546 |
| 1820907 | 1.151 | Alive | Male | 68 | 5 | 13.13 | 6.764 | 8.197 | 2.611 | 6.524 | 5.483 | 2.832 |
| 1820908 | 4.085 | Alive | Male | 53 | 2 | 11.77 | 6.234 | 6.848 | 3.432 | 5.077 | 2.797 | 2.426 |
| 1820909 | 0.647 | Dead | Male | 82 | 5 | 12.51 | 7.89 | 8.082 | 5.574 | 5.109 | 5.268 | 2.773 |
| 1820910 | 3.008 | Alive | Male | 79 | 4 | 12.01 | 6.944 | 5.785 | 2.72 | 5.263 | 7.024 | 2.38 |
| 1820911 | 0.932 | Dead | Female | 67 | 5 | 12.07 | 6.332 | 7.841 | 3.126 | 5.821 | 5.37 | 2.662 |
| 1820912 | 1.995 | Dead | Male | 79 | 7 | 14.22 | 8.344 | 8.659 | 7.795 | 6.27 | 5.714 | 3.155 |
| 1820913 | 0.364 | Dead | Male | 80 | 5 | 14.51 | 8.858 | 8.463 | 7.518 | 7.009 | 6.503 | 3.21 |
| 1820914 | 2.573 | Alive | Male | 68 | 5 | 10.02 | 2.55 | 6.274 | 4.204 | 3.552 | 5.392 | 2.158 |
