# Supplementary material for: Construction and Validation of a Novel Prognostic Signature of Idiopathic Pulmonary Fibrosis by Identifying Subtypes Based on Genes Related to 7-Methylguanosine Modification
Source: Front Genet. 2022 Jun 9;13:890530. doi: 10.3389/fgene.2022.890530 (PMC9218869; doi:10.3389/fgene.2022.890530)
Supplement: Supplementary file 9 [file Table3.DOCX]

**Supplementary Table S3.** The internal validation cohort in this study.

| ID of IPF patient | Survival  time | Status | Gender | Age | GAP | CCL2 | CCL7 | HS3ST1 | MRVI1 | TM4SF1 | TPST1 | Risk  score |
| --- | --- | --- | --- | --- | --- | --- | --- | --- | --- | --- | --- | --- |
| 1820739 | 8.016 | Alive | Male | 65 | 3 | 10.94 | 6.534 | 5.325 | 4.199 | 4.23 | 2.665 | 2.13 |
| 1820742 | 0.414 | Dead | Male | 56 | 5 | 14.46 | 10.3 | 9.557 | 8.991 | 9.283 | 10.56 | 3.614 |
| 1820761 | 1.022 | Dead | Male | 50 | 4 | 13.14 | 8.512 | 7.755 | 7.987 | 6.893 | 6.696 | 3.008 |
| 1820763 | 5.46 | Alive | Female | 77 | 2 | 11.72 | 6.315 | 8.199 | 5.38 | 4.052 | 2.655 | 2.592 |
| 1820782 | 1.466 | Dead | Female | 77 | 4 | 10.44 | 3.492 | 6.845 | 2.862 | 5.019 | 3.163 | 2.28 |
| 1820743 | 3.847 | Dead | Male | 70 | 3 | 11.07 | 6.552 | 8.081 | 5.23 | 5.632 | 3.557 | 2.63 |
| 1820746 | 2.888 | Dead | Male | 69 | 4 | 10.8 | 4.672 | 8.046 | 4.205 | 4.818 | 3.768 | 2.512 |
| 1820751 | 3.485 | Dead | Female | 74 | 4 | 9.113 | 4.93 | 5.877 | 2.605 | 5.603 | 4.699 | 2.1 |
| 1820772 | 1.184 | Dead | Male | 70 | 4 | 12.9 | 8.909 | 8.673 | 6.468 | 9.631 | 5.115 | 3.197 |
| 1820778 | 0.123 | Dead | Female | 61 | 5 | 13.45 | 9.007 | 9.039 | 6.458 | 8.543 | 6.363 | 3.247 |
| 1820779 | 1.499 | Dead | Male | 76 | 5 | 2.221 | 2.303 | 6.438 | 9.227 | 5.489 | 6.032 | 1.783 |
| 1820796 | 1.433 | Alive | Male | 69 | 8 | 10.25 | 5.692 | 7.722 | 6.029 | 7.994 | 7.929 | 2.76 |
| 1820827 | 3.025 | Alive | Male | 81 | 4 | 10.56 | 5.792 | 6.985 | 4.617 | 6.234 | 4.931 | 2.484 |
| 1820824 | 0.805 | Dead | Male | 62 | 5 | 13.19 | 7.894 | 9.138 | 6.142 | 7.61 | 7.332 | 3.18 |
| 1820748 | 0.542 | Dead | Female | 79 | 2 | 11.07 | 6.522 | 8.126 | 5.665 | 5.138 | 4.311 | 2.633 |
| 1820762 | 2.556 | Dead | Male | 67 | 7 | 12.66 | 8.36 | 8.734 | 6.61 | 7.146 | 6.063 | 3.047 |
| 1820768 | 4.847 | Dead | Male | 73 | 3 | 12.11 | 7.97 | 7.923 | 3.93 | 5.481 | 5.9 | 2.698 |
| 1820777 | 2.89 | Dead | Male | 60 | 3 | 9.513 | 5.594 | 7.041 | 5.829 | 4.798 | 2.767 | 2.308 |
| 1820786 | 0.619 | Dead | Male | 63 | 5 | 12.74 | 8.309 | 7.979 | 6.706 | 9.577 | 8.676 | 3.16 |
| 1820793 | 0.219 | Dead | Male | 67 | 7 | 16.04 | 10.92 | 9.806 | 10.02 | 9.349 | 11.73 | 3.848 |
| 1820795 | 0.411 | Dead | Female | 78 | 2 | 14.94 | 10.31 | 9.45 | 6.088 | 10.21 | 7.003 | 3.541 |
| 1820799 | 1.34 | Dead | Male | 68 | 5 | 14.33 | 9.452 | 8.812 | 6.017 | 11.88 | 7.391 | 3.511 |
| 1820811 | 2.882 | Alive | Male | 74 | 4 | 10.8 | 6.786 | 7.149 | 4.656 | 6.212 | 2.548 | 2.488 |
| 1820813 | 1.885 | Dead | Male | 76 | 5 | 13.69 | 9.792 | 9.671 | 6.159 | 9.086 | 7.602 | 3.399 |
| 1820814 | 1.351 | Dead | Male | 66 | 6 | 11.65 | 7.157 | 9.153 | 5.664 | 4.856 | 4.791 | 2.808 |
| 1820816 | 2.364 | Alive | Male | 70 | 4 | 12.1 | 6.537 | 7.616 | 5.977 | 5.46 | 4.936 | 2.701 |
| 1820817 | 1.605 | Dead | Male | 62 | 5 | 14.77 | 10.12 | 10.76 | 7.116 | 7.895 | 8.477 | 3.607 |
| 1820819 | 3.208 | Dead | Male | 70 | 4 | 10.69 | 6.07 | 6.072 | 3.93 | 7.014 | 3.939 | 2.389 |
| 1820823 | 2.304 | Alive | Female | 66 | 4 | 12.32 | 6.298 | 8.924 | 4.848 | 4.833 | 3.251 | 2.779 |
| 1820825 | 1.063 | Dead | Male | 61 | 7 | 16.6 | 12.2 | 9.434 | 10.07 | 10.26 | 9.663 | 3.877 |
| 1820830 | 0.756 | Dead | Male | 84 | 4 | 15.57 | 10.31 | 9.833 | 8.281 | 9.43 | 8.791 | 3.701 |
| 1820842 | 1.334 | Alive | Male | 59 | 5 | 12.75 | 7.639 | 9.205 | 5.829 | 5.155 | 5.003 | 2.943 |
| 1820844 | 0.773 | Dead | Male | 79 | 3 | 15.27 | 10.1 | 11.87 | 6.246 | 9.67 | 9.428 | 3.891 |
