# Supplementary material for: Construction and Validation of a Novel Prognostic Signature of Idiopathic Pulmonary Fibrosis by Identifying Subtypes Based on Genes Related to 7-Methylguanosine Modification
Source: Front Genet. 2022 Jun 9;13:890530. doi: 10.3389/fgene.2022.890530 (PMC9218869; doi:10.3389/fgene.2022.890530)
Supplement: Supplementary file 10 [file Table4.docx]

**Supplementary Table S4.** Comparison of survival status and GAP score distribution of the discovery cohort with the internal and external validation cohorts.

| Category | Discovery cohort |  | Internal validation cohort | |  | External validation cohort | |
| --- | --- | --- | --- | --- | --- | --- | --- |
|  | Sample number |  | Sample number | Chi-squared test^a^ |  | Sample number | Chi-squared test^a^ |
| 1 yr Dead | 23 |  | 9 | χ^2^ < 0.001 |  | 13 | χ^2^ = 1.024 |
| 1 yr Alive | 56 |  | 24 | *p* = 1.000 |  | 51 | *p* = 0.312 |
| 2 yr Dead | 37 |  | 18 | χ^2^ = 0.288 |  | 19 | χ^2^ = 3.674 |
| 2 yr Alive | 42 |  | 15 | *p* = 0.594 |  | 45 | *p* = 0.055 |
| 3 yr Dead | 48 |  | 21 | χ^2^ = 0.005 | | 20 | χ^2^ = 11.19 |
| 3 yr Alive | 31 |  | 12 | *p* = 0.942 |  | 44 | *p* < 0.001^*^ |
| 4 yr Dead | 50 |  | 24 | χ^2^ = 0.551 |  | 23 | χ^2^ = 9.52 |
| 4 yr Alive | 29 |  | 9 | *p* = 0.458 |  | 41 | *p* = 0.002^*^ |
| 5 yr Dead | 51 |  | 25 | χ^2^ = 0.875 |  | 24 | χ^2^ = 9.322 |
| 5 yr Alive | 28 |  | 8 | *p* = 0.350 |  | 40 | *p* = 0.002^*^ |
| GAP I | 23 |  | 8 | χ^2^ = 4.310^b^ |  | 25 | χ^2^ = 6.598^b^ |
| GAP II | 32 |  | 20 | *p* = 0.116 |  | 31 | *p* = 0.037^*^ |
| GAP III | 24 |  | 5 |  |  | 8 |  |

Notes: ^a^: Compared to the discovery cohort; ^b^: These tests are Pearson’s Chi-squared tests, while others are Pearson’s Chi-squared test with Yates’ continuity correction; ^*^*p* < 0.05.
