# Supplementary material for: Construction and Validation of a Novel Prognostic Signature of Idiopathic Pulmonary Fibrosis by Identifying Subtypes Based on Genes Related to 7-Methylguanosine Modification
Source: Front Genet. 2022 Jun 9;13:890530. doi: 10.3389/fgene.2022.890530 (PMC9218869; doi:10.3389/fgene.2022.890530)
Supplement: Supplementary file 11 [file Table6.docx]

Supplementary Table S6. The confusion matrix and F1 scores of the m7G-related prognostic signature.

| Follow-up time (year) | Predicted cases | Discovery cohort | | |  | Internal validation cohort | | |  | External validation cohort | | |
| --- | --- | --- | --- | --- | --- | --- | --- | --- | --- | --- | --- | --- |
|  |  | Actual cases | | F1 score |  | Actual cases | | F1 score |  | Actual cases | | F1 score |
|  |  | Dead | Alive |  |  | Dead | Alive |  |  | Dead | Alive |  |
| 1 | Dead | 21 | 18 | 0.677 |  | 8 | 5 | 0.727 |  | 11 | 9 | 0.667 |
|  | Alive | 2 | 38 |  |  | 1 | 19 |  |  | 2 | 42 |  |
| 2 | Dead | 30 | 9 | 0.789 |  | 13 | 0 | 0.839 |  | 12 | 6 | 0.649 |
|  | Alive | 7 | 33 |  |  | 5 | 15 |  |  | 7 | 39 |  |
| 3 | Dead | 32 | 5 | 0.753 |  | 12 | 0 | 0.727 |  | 12 | 3 | 0.686 |
|  | Alive | 16 | 26 |  |  | 9 | 12 |  |  | 8 | 41 |  |
| 4 | Dead | 35 | 0 | 0.824 |  | 11 | 0 | 0.629 |  | 11 | 0 | 0.647 |
|  | Alive | 15 | 29 |  |  | 13 | 9 |  |  | 12 | 41 |  |
| 5 | Dead | 34 | 0 | 0.800 |  | 11 | 0 | 0.611 |  |  |  |  |
|  | Alive | 17 | 28 |  |  | 14 | 8 |  |  |  |  |  |
